# Exploring the genotype-environment interaction of bread wheat in ambient and high-temperature planting conditions: a rigorous investigation

**DOI:** 10.1038/s41598-024-53052-w

**Published:** 2024-01-29

**Authors:** Vikrant Khare, Rama Shankar Shukla, Suneeta Pandey, Sanjay Kumar Singh, Charan Singh

**Affiliations:** 1 Department of Plant Breeding and Genetics, Jawaharlal Nehru Krishi Vishwavidyalaya, Jabalpur, Madhya Pradesh 482004 India; 2 Department of Plant Breeding and Genetics, Agriculture University Jodhpur, Rajasthan, 342304 India; 3 Indian Institute of Wheat and Barley Research, Karnal, Haryana 132001 India

**Keywords:** Plant sciences, Plant breeding

## Abstract

The current study is carried out to find out the stable wheat genotype in ambient and high temperature planting conditions. The objective was to estimate the genotype x environment interactions through various univariates and multivariate techniques. Twenty wheat genotypes were evaluated at Jabalpur, Narmadapuram, and Sagar districts of Madhya Pradesh, India, across cropping years 2019–20 and 2021–21, considering both timely and late planting conditions. The univariate and multivariate stability analysis were performed based on per-plant grain yield and grain filling rate. Our result revealed that environment, genotype, and GEI effects were significant (*P* < 0.001) across all the environments. The wheat genotypes JW3288, L8, and L13 have been discerned as top performers, exhibiting remarkable stability in grain yield per plant. Similarly, for grain filling rate, genotypes L11 and L13 have emerged as superior and consistently stable performers. Notably, the AMMI and GGE models demonstrated superior effectiveness and accuracy compared to the linear regression model. In conclusion, based on thorough univariate and multivariate stability analyses, L13 emerges as the most stable genotype across all environments under both planting conditions. Consequently, L13 holds promise for inclusion in future breeding programs. It's noteworthy that Jabalpur stands out as the most discriminating and representative environment among all the conditions assessed.

Wheat (*triticum aestivum L.*) is a most widely consumed cereal crop in the world^1^. It undergoes widespread cultivation on a global scale, with India assuming a pivotal role as a primary region where it serves as an indispensable staple food. In the prevailing circumstances, high temperatures have been identified as a foremost determinant in the reduction of wheat yield^2–7^. Predictable wheat yield nearly suffers from 6 to 10 percent per one ^◦^C increase in temperature at the grain-filling stage^8–10^. Rao et al.^11^ described 0.28 ^◦^C and 0.32 ^◦^C per decade increase in the least and extreme temperatures, respectively over wheat-growing parts of India. The central zone of India is determined as the most heat-prone area^12^. Nearly ~ 42%, wheat cultivating area has suffered by heat stress in the central zone of India^13^.

In earlier studies, researchers predominantly identified wheat genotypes demonstrating stable yield in the North Plain Zone of India^14–16^. Nevertheless, the exigency for a stable, heat-tolerant wheat variety is notably urgent in Central India^16^. While specific research has historically focused on the stability of wheat grain yield, recent studies have appropriately shifted their focus towards elucidating the genotype-environment interaction (GEI) for grain yield^17,18^. The meticulous control of grain yield involves a substantial number of genes with a heritability ranging from low to moderate^19^. The analysis of multi-environmental data under both heat stress and non-stress conditions, researchers sought to unravel the intricate interactions between genotype and environment for heat stress and non-stress conditions^14,20–22^.

The elucidation of genotype by environment interaction (GEI) patterns is facilitated through the application of two principal methodological approaches: regression-based models, as showcased by the frameworks established by Eberhart and Russell; and advanced multivariate stability prediction methods, exemplified by additive main effects and multiplicative interaction (AMMI), as well as genotypic main effects plus genotype by environment interaction (GGE) biplot analysis^20,23,24^. A biplot is like a special chart that helps show information from a table in two directions. It lets us see how things in rows are related and how things in columns are related at the same time. This helps us understand the connections between both rows and columns in a simple picture^25,26^. The first-time people used biplots to study farm data and pick the best model, when they looked at information from a multilocation trials of cotton^27^. After that, biplots were used to study tables that show how different types of plants interact with different environments^28^. Many plant breeders and agricultural researchers using the biplot tool because it's helpful in evaluating crop plants in different environments. Its popularity grew after it was successfully used for these purposes^29,30^. The additive main effects and multiplicative interaction (AMMI) model, characterized by its multiplicative nature, effectively gauges genotype by environment interaction (GEI) components through sophisticated multidimensional methodologies, demonstrating high discriminatory power in the estimation of such components^31,32^. Concurrently, the genotypic main effects plus genotype by environment interaction (GGE) biplot analysis serves as a valuable tool for delineating mega-environments, establishing stable genotype rankings, and pinpointing ideal varieties with consistent performance over the heat stress and non-stress conditions^16,33^. The GGE biplot was evaluated by Gauch et al.^34^ for decomposing G + G x E, while they still appealed that GGE biplots interpret G + G x E more exactly than AMMI stability^35–37^. The GGE biplot studies have been extensively used to describe macro environments, aspect genotype ranks, and correspondingly to find environments that are representative and discriminative^7^. By incorporating both main effects and interaction effects, AMMI and GGE biplots provide a comprehensive understanding of how wheat varieties respond in different climatic regimes of Central India. However, the primary objective of current study was to organize different places where plants experience stress into specific large categories called mega-environments. This helps us decide which locations affect plants in similar ways, and we can then exclude certain places from future testing. Additionally, we aim to find the best places for future tests and figure out which plant types are stable and superior in heat stress and non-stress environment. These high-quality plant types can be used as different varieties or sources for a particular mega-environment or even for adapting to a wide range of environments with heat stress tolerance.

## Materials and methods

### Experimental material

A set of 240 recombinant inbred lines was meticulously bred at the Indian Institute of Wheat and Barley Research in Karnal. This was accomplished through a meticulous crossing of the heat-susceptible parent (MACS2496) and the heat-tolerant parent (WH730), a contrast pairing that has been confirmed and validated by many researchers^5,38–41^. Among the 240 recombinant inbred lines fifteen-heat tolerant recombinant inbred lines were recognised by Department of Plant Breeding and Genetics JNKVV Jabalpur based on their performance and heat susceptibility index. Hence the identified stable recombinant inbred lines were still not registered anywhere. To further identification of stable performing recombinant inbred lines for ambient and high-temperature planting conditions. The analysis of genotype-environment interaction was meticulously conducted. To performing the analysis the selected heat tolerant recombinant inbred lines along with two parents (WH730 and MACS2496) and three commercial checks (GW322, JW3382, and JW3288), were planted at three locations (Jabalpur, Narmadapuram and Sagar), two cropping years (2019–20 and 2020–21) and two planting conditions (timely and late) of Madhya Pradesh (Table 1 and Supplemental Table s1 and s2).

**Table 1:** Pedigree and heat susceptibility indices of studied wheat genotypes.

Genotypes	Y_(hsi)_	Pedigree	Origin
*Heat tolerant recombinant inbred lines*			
L1	0.03	MACS2496/WH730	IIWBR, Karnal
L2	0.12	MACS2496/WH730	IIWBR, Karnal
L3	0.21	MACS2496/WH730	IIWBR, Karnal
L4	0.23	MACS2496/WH730	IIWBR, Karnal
L5	0.25	MACS2496/WH730	IIWBR, Karnal
L6	0.28	MACS2496/WH730	IIWBR, Karnal
L7	0.28	MACS2496/WH730	IIWBR, Karnal
L8	0.37	MACS2496/WH730	IIWBR, Karnal
L9	0.38	MACS2496/WH730	IIWBR, Karnal
L10	0.38	MACS2496/WH730	IIWBR, Karnal
L11	0.41	MACS2496/WH730	IIWBR, Karnal
L12	0.42	MACS2496/WH730	IIWBR, Karnal
L13	0.43	MACS2496/WH730	IIWBR, Karnal
L14	0.44	MACS2496/WH730	IIWBR, Karnal
L15	0.44	MACS2496/WH730	IIWBR, Karnal
*Parents*			
WH730	0.45	CPAN2092/Improved Lok 1	HAU, Hisar
MACS2496	1.7	SERI "S"	ARI, Pune
*Commercial checks*			
GW322	1.69	GW173/GW196	SDAU, VIJAPUR
JW3382	0.66	CHOIX/STAR/3/HE1/3*CNO79//2*SERI/4/GW273	JNKVV, Jabalpur
JW3288	1.24	DOVE/BUC/DL 788–2	JNKVV, Jabalpur

*Y*_*(hsi)*_ = heat susceptibility index based on grain yield.

### Testing environment

Selected heat-tolerant recombinant inbred lines, their parents and commercial checks were tested into timely sown and late sown planting conditions. Timely sown environments designated as NSE (normal sown environment) and late sown environment designated as HSE (heat stressed environment). Under NSE the experimental material was screened in two cropping years (2019–20 and 2020–21) at three locations (Jabalpur, Sagar and Narmadapuram) on the other hand under HSE the experimental material was screened in two cropping years (2019–20 and 2020–21) at two locations (Jabalpur and Narmadapuram). The maximum and minimum day—night temperatures during two cropping years (2019–20 and 2020–21), under NSE, 36.8/16.7–21.4/4.8 °C, 39.1/22.8–23.1/02.1 °C and 39.1/22.8–23.1/02.1 °C, were recorded at Jabalpur, Sagar and Narmadapuram, respectively while under HSE, 39.3/23.2–22/4.8 °C and 41.5/12.2–20.5/2.9 °C, were recorded at Jabalpur and Narmadapuram, respectively (Supplemental Table s5, s6 and s7). Geographically the experimental field of Jabalpur, Sagar and Narmadapuram were located at 23.21, 23.83, and 21.50, latitudes (N) 79.95, 78.71, and 76.43, longitudes (E) and heights from mean sea level were recorded 392, 433, and 229 m, respectively. Geographically, JNKVV's College of Agriculture Jabalpur, Regional Agriculture Research Centre Sagar, and College of Agriculture Narmadapuram were located at latitudes (N) of 23.21, 23.83, and 21.50, longitudes (E) of 79.95, 78.71, and 76.43, and heights(m) from mean sea level of 392, 433, and 229, respectively (Table 2). For the experimental fields in Jabalpur, Sagar, and Narmadapuram, the soil pH values were 7.61, 6.96, and 8.10, respectively (Table 2). Throughout the cropping period, adherence to recommended packages and practices, coupled with the implementation of an optimal number of irrigations, was rigorously maintained.

**Table 2:** Experimental details.

General detail of experimental locations					
Environment	Locations	Planting year	Planting Date	Day night temperature range (°C)	
NSE1	Jabalpur	2019–20	2^nd^ Dec	36.8/20.4—21.0/6.3	
NSE2	Sagar	2019–20	2^nd^ Dec	39.1/22.8—19.5/5.8	
NSE3	Narmadapuram	2019–20	2^nd^ Dec	37.6/14.6—17.1/3.1	
NSE4	Jabalpur	2020–21	2^nd^ Dec	36.3/16.7—21.4/4.8	
NSE5	Sagar	2020–21	2^nd^ Dec	38.1/23.5—23.1/2.1	
NSE6	Narmadapuram	2020–21	2^nd^ Dec	36.1/22.5—12.5/4.2	
HSE1	Jabalpur	2019–20	4^th^ Jan	39.3/24.0—22.0/7.5	
HSE2	Narmadapuram	2019–20	4^th^ Jan	41.5/12.2—20.2/2.9	
HSE3	Jabalpur	2020–21	4^th^ Jan	38.8/23.2—21.4/4.8	
HSE4	Narmadapuram	2020–21	4^th^ Jan	40.3/24.5—20.5/4.2	

Geographical detail of experimental locations					
Latitude (N)	Longitude (E)	Altitude (a.m.s.l.)	Soil Colour	Soil pH	Locations
23.21	79.95	392	Dark grey	7.61	Jabalpur
23.83	78.71	433	Black soils	6.96	Sagar
21.5	76.43	229	Deep black soil	8.1	Narmadapuram

*N* = North, *E* = East, *a.m.s.l*. = Above mean sea level, *NSE* = normal sown environment, *HSE* = Heat stress environment, *Dec* = December, *Jan* = January.

### Design of experiment and data collections

Each experimental trial was conducted in randomised block design with three replications. Numerous component traits were observed manually, including days to heading, days to maturity, thousand kernel weight, grain filling duration, grain filling rate and grain yield per plant. Grain filling rate and grain yield per plant were subjected to the current study. As per Dias and Lidon's^42^ methodology, the comprehensive span from anthesis to maturity was considered as the grain filling duration. This duration was subsequently utilized to calculate the grain filling rate in grams per day using the formula (total grain yield per plant/grain filling duration).

### Statistical analysis

#### Analysis of variance and association study

Fischer and Maurer's^43^ method, 1-(y_s_/y_p_)/1-(x_s_/x_p_), was used to estimate the heat susceptibility index (HSI). Y_s_ stands for yield under stress, Y_p_ for yield without stress, and X_s_ and X_p_ stand for mean yields across all cultivars under stress and non-stress circumstances, respectively. "Stress intensity" is the definition of the expression (1- X_s_/X_p_). To ascertain the variance among grain yield per plant and grain filling rate, a combined ANOVA was performed through various packages of R software version 4.2. Whereas genotypes were measured as fixed factors, environments were measured as random variables. The "corroplot" package of R software was used to perform the pearson association between studied traits and heat susceptibility index, and the resulting model is given as: rG = cov (A, B)/var(A), var(B), where cov (A, B) designates the covariance present among traits, and var (A), and similarly, var (B), displays the genetic alteration of trait^44^.

#### Regression based stability analysis

Initially genotype x environment interactions were estimated through regression-based stability models. Eberhart and Russell's model^45^, the regression coefficient (*b*_*i*_), and the deviation from regression (*S*^*2*^*d*_*i*_) are all stability indicators based on regression. These parameters collectively govern the performance of a genotype across a variety of contexts^46^. The Eberhart and Russell’s stability model^45^ is given as: Y_ij_ = μ_i_ + β_i_I_j_ + δ_ij_, where the Y_ij_ designates the assessment of ith (i = 1, 2, 3, . . .. . ., x) genotype across the *j*th (1, 2, 3, . . .. . ., n) environment, μ_i_ is the genotype mean, β_i_ designates the regression coefficient, δ_ij_ demonstrations the deviation from the regression coefficient, and I_j_ is the environmental index acknowledged by deducting the total mean from each environmental mean^47^.

#### AMMI and GGE biplot analysis

Additionally, the multivariate method for stability analysis were directed rendering to AMMI and GGE biplot by means of different statistical packages obtainable in R studio. The “metan” package of R studio was practical for AMMI analysis, while the GGE Biplot GUI package was working for GGE biplot based analysis. In the AMMI model, the ANOVA and PCA are compound collected into an individual statistical package. Therefore, GEI exposed to PCA investigation only when key authentication has already been accomplished by means of ANOVA^48^. The equation for AMMI model is specified as below: Y_ge_ = μ + α_g_ + β_e_ + Σ_n_λ_n_γ_gn_δ_en_ + ρ_ge_, where in circumstance of the additive factors, Y_ge_ is display the grain yield and grain filling rate for a specific (g) genotype in an (e) environment, where μ stand for grand mean, α_g_ designates deviation of genotype from the mean, β_e_ is deviation of environment from the mean, λ_n_ stands for singular value of n component, γ_gn_ designates the value of eigenvector for genotype (g) and δ_en_ is the value of eigenvector for e and ρ_ge_; which is known as residual^49^. The AMMI stability values (ASV) were estimated as per Rad et al.^49^, ASV = √[(SSIPCA1/SSIPCA2) (IPCA1)^2^] + (IPCA2)^2^, Where, SSIPCA1 and SSIPCA2 are sum of squares of IPCA1 and IPCA2, respectively and IPCA1 and IPCA2 are the genotypic scores in the AMMI model. Moreover, the equation for GGE biplot model is characterized as: P_ij_ = (y_ij_—μ—δ_j_)/λ_j_ = (β_i_ + ϵ_ij_)/λ_j_, where P_ij_ is the matrix for genotype i and environment j, μ denotes the grand mean, δ_j_ is the column (environment) main effect, λ_j_ is an evaluating factor, β_i_ is the row (genotype) main effect, and ϵ_ij_ characterizes genotype x environment interaction, and y_ij_ is genotype and environment, two-way table^50^. The GGE biplot also includes a group of biplot-based platforms for interpreting interactions between the environment and the genotype. In a general context, for addressing inquiries related to Genotype by Environment (G x E), both GGE biplot and AMMI utilize graphical representations^51^. In total, the results of both biplot analyses are further interpreted based on the standards established by Khan et al^15^.The Genomic Selection Index (GSI) was computed following Farshadfar's methodology^43^, where GSI is defined as the sum of the rank of ASV (R_ASV_) and the mean grain yield rank of the genotype (R_gm_).

### Bioethical statement

The seed material remains unregistered and has not been submitted to any publicly accessible herbarium. It was acquired from IIWBR, Karnal, through a legally binding agreement, without any associated costs. We emphasize our unwavering commitment to strict adherence to all local, national, and international guidelines and legislation governing the use of plants in this study, as delineated in the editorial policies for research involving plants (https://www.nature.com/srep/journal-policies/editorialpolicies#research-involving-plants).

## Results

### Combined analysis of variance

The pooled analysis of variance (ANOVA) was used to identify interactions between and within the sources of variation that were examined in this investigation. Table 3 contains the results of the combined ANOVA for grain yield and grain filling rate. Under normal and heat stress environment, omitting the year for grain filling rate under heat stress, replication for grain yield under normal and heat stress environment, the variation owing to genotype (G), location (L), year (Y), G x Y, G x L, and G x Y x L interactions for all two characters was significant, either at 0.001% or 0.05% level of significance. For grain yield and grain filling rate, wheat genotypes shown a considerable degree of heterogeneity.

**Table 3:** Combined analysis of variances for wheat genotypes under normal and heat stress environment.

Sources	Normal sown environment (NSE)			Heat stress environment (HSE)		
	DF	YLD	GFR	DF	YLD	GFR
Genotype (G)	19	57.81***	0.078***	19	42.97***	0.014***
Replication (R)	2	0.82^ ns^	0.001*	2	0.0001^ ns^	0.006*
Location (L)	2	23.78***	0.281***	1	34.77***	0.059***
Year (Y)	1	55.36***	0.005*	1	13.45***	0.001^ ns^
G x L	38	16.81***	0.579***	19	15.89***	0.014***
G x Y	19	15.55***	0.013***	19	26.57***	0.007***
L x Y	2	14.78***	0.043***	1	33.89***	0.041***
G x L x Y	38	15.44***	0.012***	19	22.84***	0.008***
Residual	120	0.851	0.111	80	0.317	0.001

*DF* = degree of freedom, *YLD* = grain yield, *GFR* = grain filling rate, *G x L* = Genotype x Location, *G x Y* = Genotype x Year, *L x Y* = Location x Year, *G x L x Y* = Genotype x Location x Year, *P < 0.05, ***P < 0.001, *ns* = non-significant.

### Association study

Supplemental Figure s1 illustrates a highly significant correlation (*P* = 0.001%) between grain filling rate and heat susceptibility index with grain yield. The strong positive and negative correlation originated among grain yield in normal sown environment (*r* = 0.80) and heat stress environment (*r* = − 0.82) with heat susceptibility index, respectively. The grain filling rate (*r* = 0.79) was found significance (*P* = 0.001%)) strong and positive correlation with grain yield.

### Regression based stability analysis

The slope of linear regression (*b*_*i*_) and deviation from regression (*S*^2^*d*_*i*_) displayed a vast range, for heat tolerant wheat genotypes (Tables 4 and 5). Across normal sown environments, *b*_*i*_ for grain yield ranged from 0.22 (L2) to 1.76 (L5), and absolute values of *S*^2^*d*_*i*_ ranged from 0.35 (L12) to 11.20 (WH730) while for grain filling rate *b*_*i*_ range from 0.05 (L9) to 2.65 (WH730), and absolute value of *s*^2^*d*_*i*_ ranged from zero (L11) to 0.027 (WH730) (Table 4). On the other hand, across heat stress environments for grain yield, *b*_*i*_ ranged from − 1.91 (L10) to 3.13 (MACS2496), with the absolute values of *S*^2^*d*_*i*_ranging from − 0.05 (L15) to 12.4 (L5) (Table 5). Among the wheat genotypes for grain yield, L12 (*S*^2^*d*_*i*_ = 0.35) and L13 (*S*^2^*d*_*i*_ = 0.84) under normal sown environment, L15 (*S*^2^*d*_*i*_ =  − 0.05) and L9 (*S*^2^*d*_*i*_ = 0.25) under heat stress environment showed lower *S*^2^*d*_*i*_value (Tables 4 and 5). Similarly, for grain filling rate, L11 (*S*^2^*d*_*i*_ = 0.0) under normal sown environment while under heat stress environment WH730, MACS2496 and GW322 (0.0) showed lower *S*^2^*d*_*i*_ value (Tables 4 and 5). Under normal sown environments (NSE) L12 (*b*_*i*_ = 1.44, *S*^2^*d*_*i*_ = 0.35) for grain yield and L11 (*b*_*i*_ = 1.14, *S*^2^*d*_*i*_ = 0.0) for grain filling rate were identified as most stable genotypes (Table 4). For grain yield L3 (*b*_*i*_ = 0.79, *S*^2^*d*_*i*_ = 0.18) and for grain filling rate L6 (*b*_*i*_ = 0.85, *S*^2^*d*_*i*_ = 0.007) were identified as most stable wheat genotype under heat stress environments (HSE) (Table 5). Across all the environments, good levels of stability for grain yield were exhibited by WH730 (*b*_*i*_ = 0.78, *S*^2^*d*_*i*_ = 0.05), while for grain filling rate L6 (*b*_*i*_ = 0.97, *S*^2^*d*_*i*_ = 0.0) followed by JW3382 (*b*_*i*_ = 0.93, *S*^2^*d*_*i*_ = 0.0) were found most stable genotype (Supplemental Table s3).

**Table 4:** Stability parameters for grain yield and grain filling rate under normal sown environment.

Genotype	Grain yield							Grain filling rate						
	G_m_	R_gm_	*b*_*i*_	*S*^*2*^*d*_*i*_	ASV	R_ASV_	GSI	G_m_	R_gm_	*b*_*i*_	*S*^*2*^*d*_*i*_	ASV	R_ASV_	GSI
*Heat tolerant recombinant inbred lines*														
L1	11.38	16	0.64	2.95	1.31	7	23	0.30	16	0.18	0.001	0.05	1	17
L2	10.75	19	0.22	4.88	2.32	11	30	0.29	17	0.33	0.006	0.47	13	30
L3	10.65	20	0.29	8.71	1.87	9	29	0.31	14	0.88	0.012	0.62	17	31
L4	12.37	12	0.63	0.89	0.46	2	14	0.28	19	0.87	0.002	0.27	9	28
L5	12.98	7	1.76	9.42	3.90	17	24	0.41	4	1.98	0.006	0.79	18	22
L6	12.13	13	0.98	4.11	1.70	8	21	0.29	18	0.69	0.004	0.20	6	24
L7	11.18	17	1.57	5.16	2.53	12	29	0.31	13	1.49	0.004	0.25	8	21
L8	12.63	9	0.30	7.34	3.52	16	25	0.31	12	0.20	0.003	0.55	14	26
L9	10.91	18	0.45	10.3	4.21	19	37	0.30	15	0.05	0.009	0.83	19	34
L10	11.93	15	0.85	5.17	2.65	13	28	0.32	11	0.83	0.004	0.43	12	23
L11	13.74	6	1.42	2.96	1.18	5	11	0.39	6	1.14	0.000	0.07	2	8
L12	12.57	10	1.44	0.35	0.41	1	11	0.33	10	1.47	0.001	0.21	7	17
L13	12.92	8	1.48	0.84	0.74	3	11	0.39	7	1.55	0.005	0.28	10	17
L14	12.38	11	0.91	6.35	2.68	15	26	0.36	9	0.73	0.007	0.58	16	25
L15	11.96	14	0.75	6.11	2.68	14	28	0.27	20	0.84	0.001	0.20	5	25
*Parents*														
WH730	19.10	1	1.74	11.20	5.95	20	21	0.62	1	2.65	0.027	1.55	20	21
MACS2496	15.67	2	1.65	10.60	3.98	18	20	0.44	2	1.62	0.006	0.31	11	13
*Commercial checks*														
GW322	14.47	5	1.33	3.73	1.26	6	11	0.40	5	1.47	0.001	0.18	4	9
JW3382	14.83	3	1.07	8.67	1.96	10	13	0.38	8	0.97	0.005	0.11	3	11
JW3288	14.59	4	0.51	3.58	1.10	4	8	0.43	3	0.06	0.009	0.56	15	18

*G*_*m* =_ Grand mean, *R*_*gm*_ = Rank of genotypes based grand mean*, b*_i_ = Regression coefficient of Eberhart and Russell model*, S*^*2*^*d*_*i*_ = Deviation form regression of Eberhart and Russel model*, ASV* = AMMI stability value, *R*_*ASV*_ = Stability rank based on AMMI stability value, *GSI* = Genomic selection index.

**Table 5:** Stability parameters for grain yield and grain filling rate under heat stress environment.

Genotypes	Grain yield							Grain filling rate						
	G_m_	R_gm_	*b*_*i*_	*S*^*2*^*d*_*i*_	ASV	R_ASV_	GSI	*G*_*m*_	R_gm_	*b*_*i*_	*S*^*2*^*d*_*i*_	ASV	R_ASV_	GSI
*Heat tolerant recombinant inbred lines*														
L1	11.29	10	2.44	0.97	0.498	4	14	0.366	17	0.61	0.002	0.2	7	24
L2	10.45	17	− 0.7	1.34	0.911	12	29	0.352	18	− 1.58	0.001	0.5	17	35
L3	9.76	18	0.79	0.18	0.323	2	20	0.423	9	2.76	0.003	0.42	15	24
L4	11.66	5	1.8	6.4	1.458	17	22	0.424	8	2.04	0.001	0.16	5	13
L5	11.88	3	0.52	12.4	2.214	20	23	0.469	2	0.64	0.006	0.24	9	11
L6	11.01	14	0.61	3.51	1.017	13	27	0.382	16	0.85	0.007	0.06	1	17
L7	10.48	16	1.83	1.03	0.778	11	27	0.456	5	1.95	0.001	0.27	11	16
L8	11.99	2	0.72	1.29	0.66	7	9	0.347	20	0.61	0.001	0.06	2	22
L9	9.7	20	1.75	0.25	0.499	5	25	0.351	19	1.9	0.001	0.21	8	27
L10	11.19	13	− 1.91	3.14	0.726	9	22	0.476	1	1.29	0.02	0.51	18	19
L11	12.29	1	− 0.41	6.47	1.47	18	19	0.429	7	− 0.82	0.004	0.34	12	19
L12	11.5	7	− 0.31	6.18	1.61	19	26	0.461	4	− 1.54	0.003	0.54	20	24
L13	11.54	6	1.48	1.49	0.693	8	14	0.454	6	0.51	0.003	0.11	3	9
L14	11.2	12	2.27	0.92	0.607	6	18	0.467	3	2.66	0.004	0.25	10	13
L15	11.4	9	0.04	− 0.05	0.115	1	10	0.385	14	− 1.29	0.001	0.44	16	30
*Parents*														
WH730	11.84	4	2.59	3.35	1.31	16	20	0.414	11	3.33	0	0.4	14	25
MACS2496	9.71	19	3.13	1.81	1.056	14	33	0.39	13	3.97	0	0.52	19	32
*Commercial checks*														
GW322	11.23	11	− 0.87	0.89	0.426	3	14	0.414	12	− 1.46	0	0.39	13	25
JW3382	11.44	8	1.36	1.33	0.75	10	18	0.416	10	1.46	0.002	0.15	4	14
JW3288	10.53	15	2.85	3.63	1.291	15	30	0.385	15	2.09	0.004	0.2	6	21

*G*_*m* =_ Grand mean, *R*_*gm*_ = Rank of genotypes based grand mean*, b*_*i*_ = Regression coefficient of Eberhart and Russell model*, S*^*2*^*d*_*i*_ = Deviation form regression of Eberhart and Russel model*, ASV* = AMMI stability value, *R*_*ASV*_ = Stability rank based on AMMI stability value, *GSI* = Genomic selection index.

### Additive main effect and multiplicative interactions (AMMI) analysis

#### AMMI based ANOVA

The AMMI-based analysis of variance (ANOVA) results for the current study's evaluations of grain yield and grain filling rate are shown in Table 6 and supplemental table s4. The results showed that G, E, and GEI significantly influenced by grain yield and grain filling rate. The first two interaction principal component analysis (IPCA) axes explained about 68.9, 78.2 and 68.9% of the GEI across normal sown, heat stressed and across all the environments for grain yield, respectively while 68.9, 78.2 and 59.5% of GEI were explained by first two IPCA for grain filling rate in normal sown, heat stress and across all the environments, respectively (Table 6 and supplemental table s4). Across all the environments proportion of total variation contributed by G, E, and GEI were executed for grain yield was 12.8, 12.3, and 35.8, percentage, respectively while for grain filling rate 15.4, 14.5 and 33.7 percentage, respectively (Supplemental table s4).

**Table 6:** AMMI-based ANOVA for grain yield and grain filling rate.

Source	DF	Grain yield			Grain filling rate		
		MSS	Percent explained	*p *value	MSS	Percent explained	*p *value
*Under normal sown environment*							
Environment	5	132.08	15.4	< 0.001	0.131	13.54	< 0.001
Block	6	1.14	0.2	0.234	0	0.03	0.941
Genotype	19	49.17	21.8	< 0.001	0.079	30.97	< 0.001
G x E	95	13.61	30.2	< 0.001	0.014	26.58	< 0.001
PC1	23	32.3	57.4	< 0.001	0.029	52.2	< 0.001
PC2	21	13.37	21.7	< 0.001	0.01	16.7	< 0.001
PC3	19	10.07	14.8	< 0.001	0.01	14.2	< 0.001
PC4	17	4.62	6.1	< 0.001	0.008	10.2	< 0.001
PC5	15	0	0	< 0.001	0.006	6.7	< 0.001
Residuals	114	0.84	-	-	0.001	-	-
Total	334	12.82	-	-	0.014	-	-
*Under heat stress environment*							
Environment	3	12.219	9.74	< 0.001	0.033	6.5	0.02
Block	4	0.806	0.42	0.082	0.003	0.77	0.009
Genotype	19	4.677	19.5	< 0.001	0.014	17.35	< 0.001
G x E	57	5.401	39.83	< 0.001	0.01	35.69	< 0.001
PC1	21	6.722	45.9	< 0.001	0.014	54.3	< 0.001
PC2	19	5.635	34.8	< 0.001	0.007	23.9	< 0.001
PC3	17	3.508	19.4	< 0.001	0.007	21.8	< 0.001
Residuals	76	0.374	-	-	0.001	-	-
Total	216	3.578	-	-	0.007	-	-

*DF* = Degree of freedom, *MSS* = Means of sum squire, *G x E* = Genotype x Environment and *PC* = Principal component.

#### Stable genotypes identified based on AMMI-ASV and GSI value

The AMMI, additive main effects and multiplicative interaction stability value (ASV) is presented for grain yield and grain filling rate under normal sown, heat stress and across all the environments in the Tables 4, 5 and supplemental table s3. The range of ASV value from 0.41 (L12) to 5.95 (WH730) and 0.05 (L1) to 1.55 (WH730) were observed for grain yield and grain filling rate respectively, in normal sown environments (Table 4). Similarly, the range of ASV value from 0.115 (L15) to 2.21 (L5) and 0.06 (L6) to 0.54 (L12) were observed for grain yield and grain filling rate respectively, in heat stress environments (Table 5). For grain yield L12 (ASV = 0.41) in normal sown, L15 (ASV = 0.41) in heat stress and WH730 (ASV = 0.20) across all the environments while for grain filling rate L1 (ASV = 0.05) in normal sown, L6 (ASV = 0.06) in heat stress and L1 (ASV = 0.02) across all the environments were observed as most stable genotypes (Tables 4, 5 and supplemental table s3). The genomic selection index was estimated and presented for grain yield and grain filling rate in Table 4, 5 and supplemental table s3. Genotype with the lowest stability parameter was deemed stable, and that with a lower GSI value had higher mean yield and higher stability. For grain yield the JW3288 (GSI rank = 8) in normal sown, L8 (GSI rank = 9) in heat stress and L13 (GSI rank = 9) in all the environments while for grain filling rate L11 (GSI rank = 8) in normal sown, L13 (GSI rank = 9) in heat stress and GSI rank = 9 was achieved by L11 and L13 in all the environments, suggested that high stable and well performed genotypes (Table 4, 5 and supplemental table s3).

#### AMMI 1

**Figure 1:**
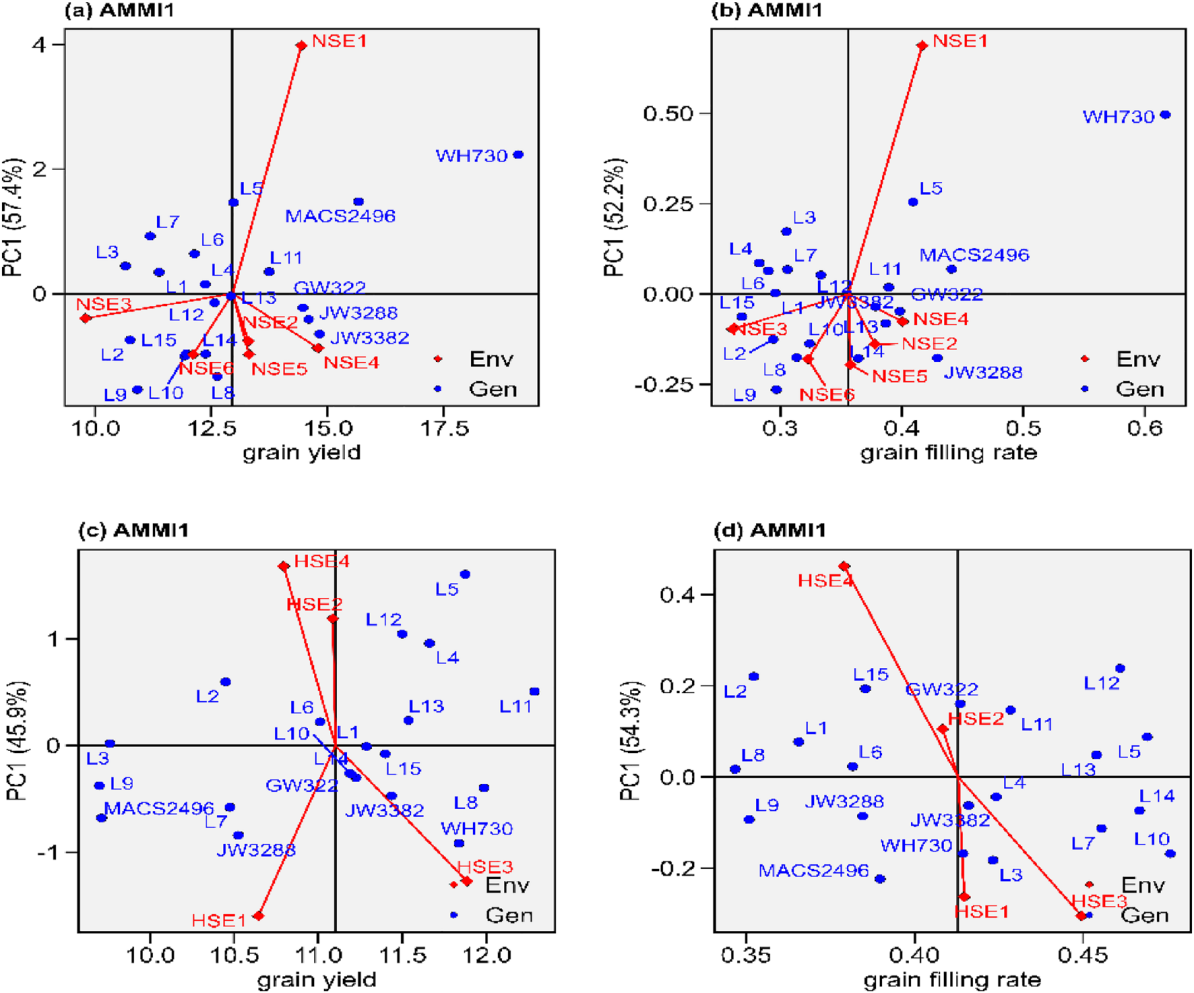
The “AMMI1” graphs displays the main effect and IPC1 effect values describing relationship among examined wheat genotypes and environments. (**a**) grain yield under normal sown environment, (**b**) grain filling rate under normal sown environment, (**c**) grain yield under heat stress environment (**d**) grain filling rate under heat stress environment.

**Figure 2:**
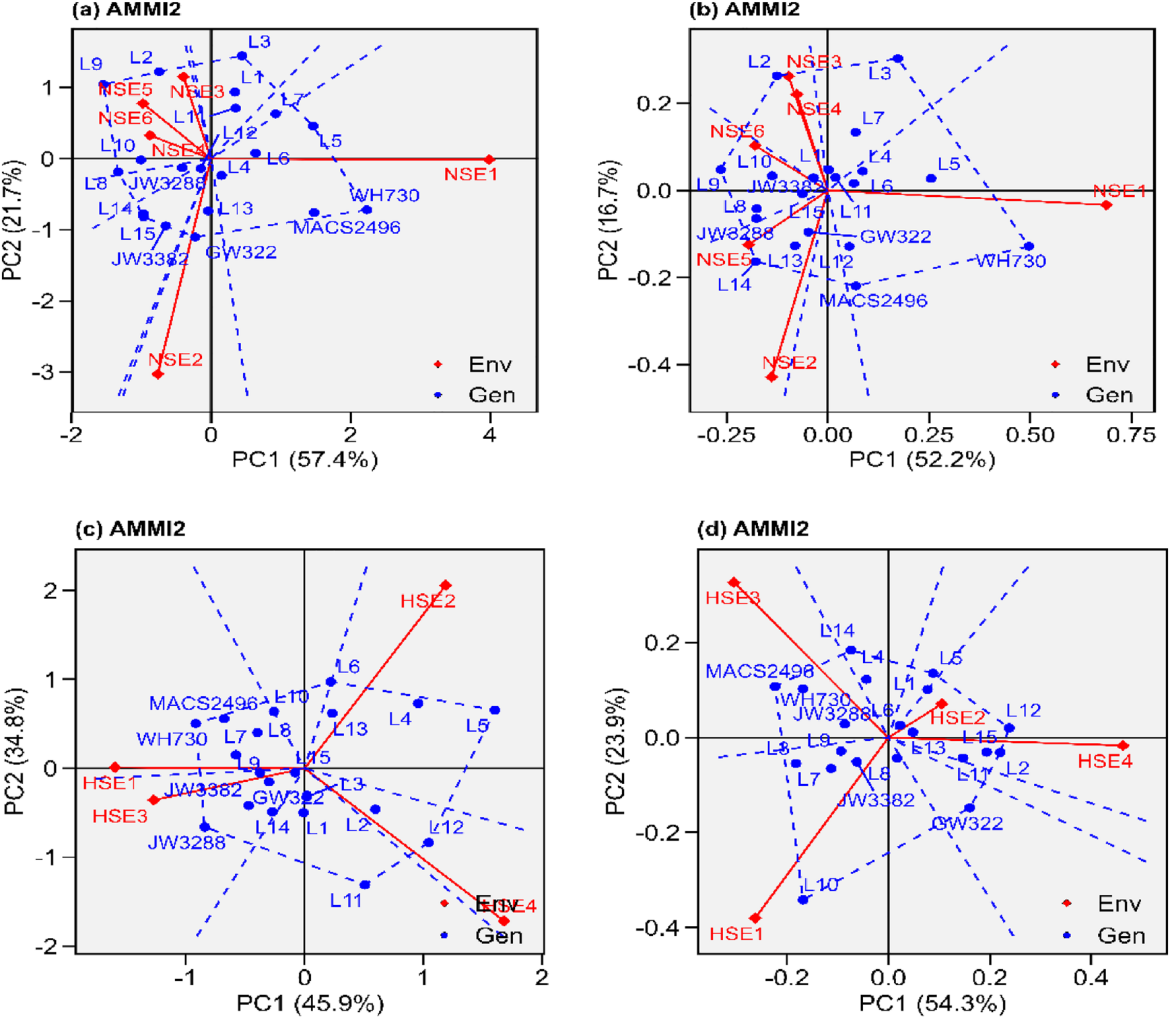
“AMMI2” graphs displays both the axes of interaction (IPCA1 and IPCA2) values for genotype effect and genotype by environment interaction effect. (**a**) grain yield under normal sown environment, (**b**) grain filling rate under normal sown environment, (**c**) grain yield under heat stress environment (**d**) grain filling rate under heat stress environment.

The AMMI biplots of 20 (Supplemental table s1 and s2) heat stress tolerance wheat genotypes across normal sown, heat stress sown and across both the environmental conditions are presented in Figs. 1, 2, supplemental fig. s2 and s3. In the current study, IPCA1 for grain yield explained 57.4, 45.9, and 46.2% while for grain filling rate 52.2, 54.3 and 39.6% of the variation attributed to GEI in normal sown, heat stress and across both the environmental conditions, respectively (Fig. 1. and supplemental fig. s2). Based on AAMI1, for grain yield NSE4 and HSE3 (Fig. 1a and c) while for grain filling rate NSE1 and HSE3 (Fig. 1b and d) are high-potential environments under normal sown and heat stress environments, respectively. Similarly for grain yield NSE3 and HSE1 (Fig. 1a and b) while for grain filling rate NSE3 and HSE4 (Fig. 1c and d) are low-potential environments under normal sown and heat stress environments, respectively. Similarly, for grain yield and grain filling rate, WH730 are specifically adapted to high-potential environments in normal sown environments while respectively, for grain yield and grain filling rate L11 and L10 are specifically adapted to high-potential environments in heat stress environments, (Fig. 1.). With an almost zero IPCA1 score, the cultivar is said to have a poor interaction with its environment it means the cultivar have high stability. For grain yield and grain filling rate NSE3 (Fig. 1a and b) across normal sown environments while HSE2 (Fig. 1b and d) across heat stress environments had a PCA1 score or vector nearer to zero compared to other environments, specifies lower interaction effect which nearly ensures the better performance of all genotypes in that environment. Hence, for grain yield L1, L13 and GW322 (Fig. 1a) while for grain filling rate L6, L12, L11 and GW322 (Fig. 1b) might be the utmost stable genotypes among the normal sown environments based on IPCA1. Similarly, for grain yield L3, L1 and L15 (Fig. 1c) while for grain filling rate L8, L6, L4 and L13 (Fig. 1d) identified as most stable genotype among heat stress environment. Across all the environments for grain yield and grain filling rate, NSE3 and NSE6 identified as most stable environment respectively, on the other hand, for grain yield L13, L1, L12, L13 and GW322 while for grain filling rate L1, L6, JW3382 and GW322 identified as most stable genotype across all the environments (Supplemental fig. s1).

#### AMMI 2

The AMMI2 expose and inferring the difficult GEI that comprises significant multi-environments and finding of genotypes with also broad or narrow spectrum adaptability. For grain yield and grain filling rate under normal sown environments, NSE1 (Fig. 2a and b) while under heat stressed environments, HSE4 for grain yield and HSE1 for grain filing rate was farthest from the origin, suggesting that the best discriminatory ability but was not stable. In contrast, for grain yield NSE4 (Fig. 2a) and for grain filling rate NSE6 (Fig. 2b), across normal sown environments while for grain yield HSE3 (Fig. 2c) and for grain filling rate HSE2 (Fig. 2d) was nearest to the origin, suggesting that was the most stable environment across heat stressed environments. Across all the tested environments NSE3 and NSE4 were very closer to the origin suggested as most stable environments for grain yield and grain filling rate, respectively (Supplemental fig. s2). In contrast to genotypes that are placed apart from one another, genotypes that assemble together on the biplot origin suggested that genotypes have identical response to all tested environment. Also, compared to genotypes that are near positioned to the biplot origin, those that are located away are more vulnerable to environmental interactions.

### GGE Biplot analysis

**Figure 3:**
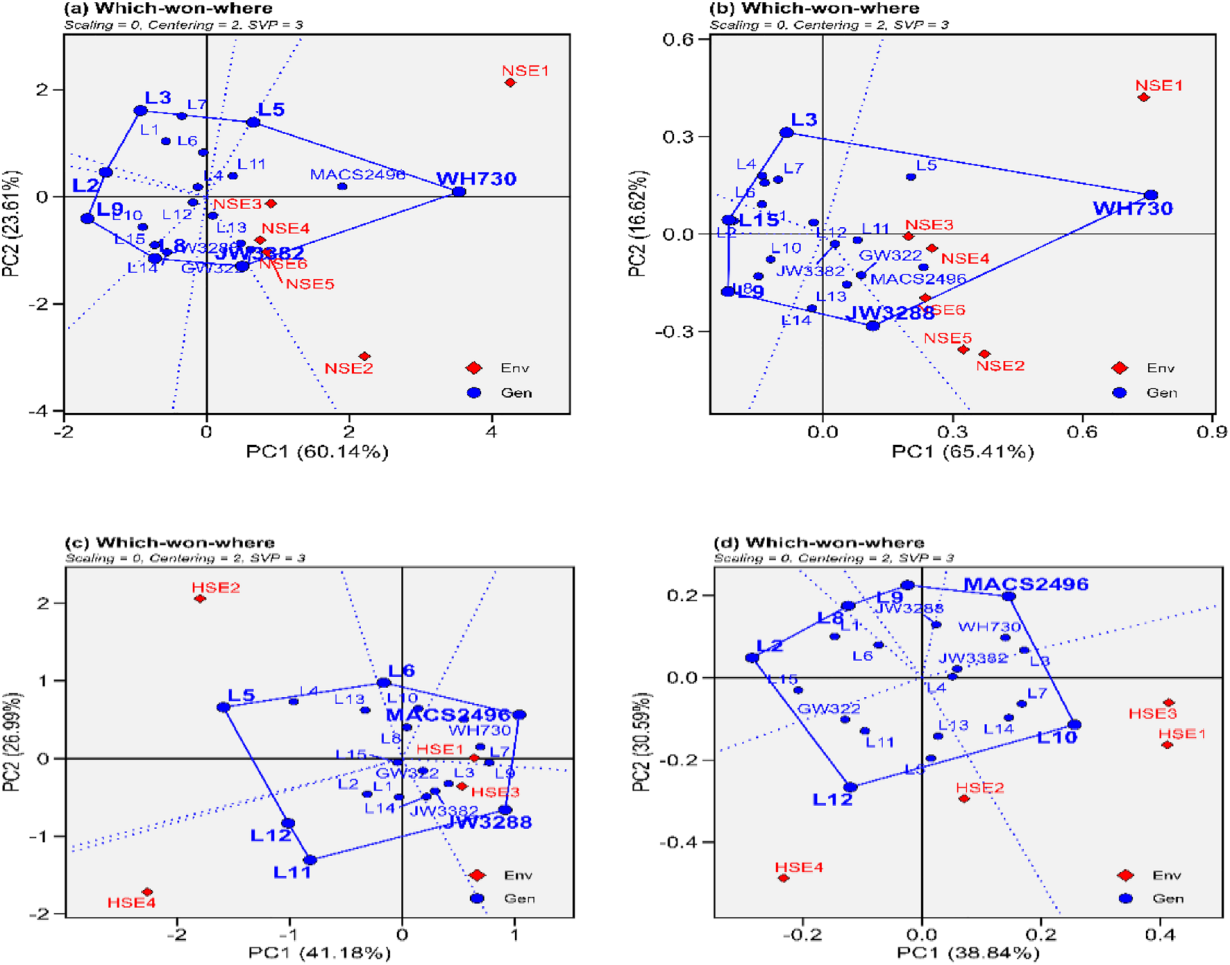
The polygon view of “Which-won-where” model of GGE biplot representing the performance of wheat genotypes and their interactions with environment. (**a**) grain yield under normal sown environment, (**b**) grain filling rate under normal sown environment, (**c**) grain yield under heat stress environment (**d**) grain filling rate under heat stress environment.

**Figure 4:**
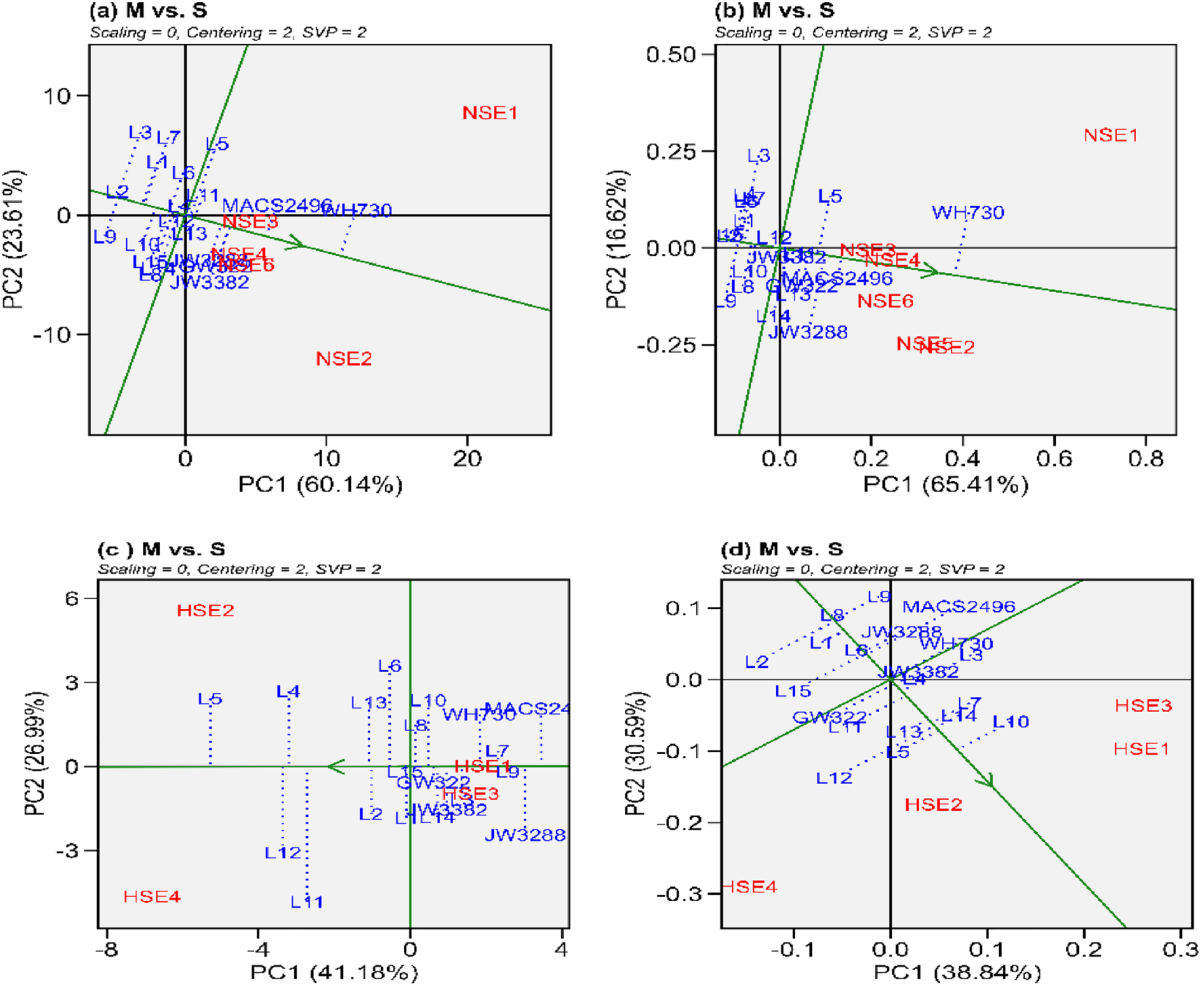
The “mean versus stability” model describe the interaction effect of wheat genotypes. (**a**) grain yield under normal sown environment, (**b**) grain filling rate under normal sown environment, (**c**) grain yield under heat stress environment (**d**) grain filling rate under heat stress environment.

**Figure 5:**
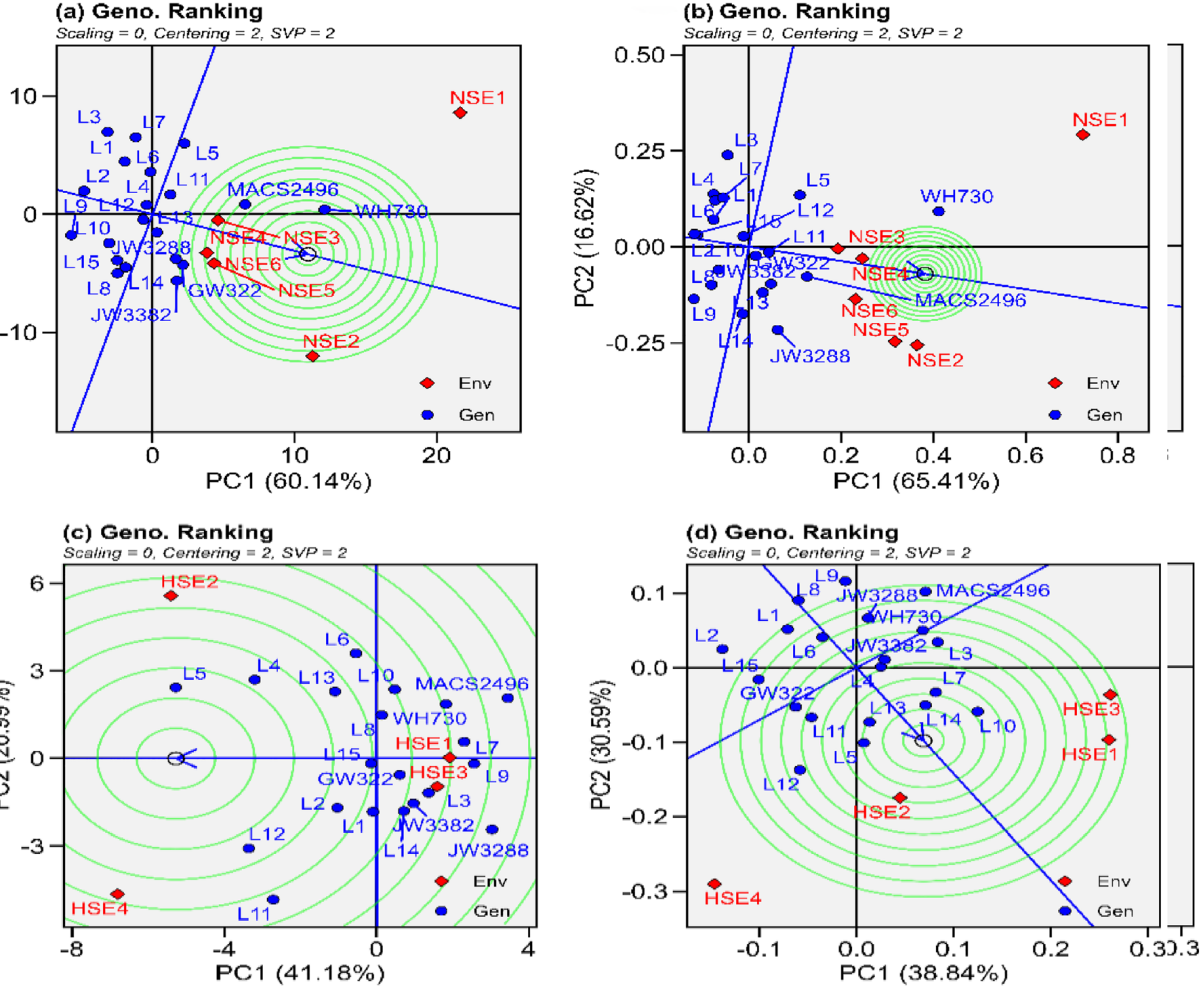
“Ranking of genotypes” model of biplot assess other wheat genotypes against the ideal genotype conferring genotype interaction and genotype x environment interactions. (**a**) grain yield under normal sown environment, (**b**) grain filling rate under normal sown environment, (**c**) grain yield under heat stress environment (**d**) grain filling rate under heat stress environment.

**Figure 6:**
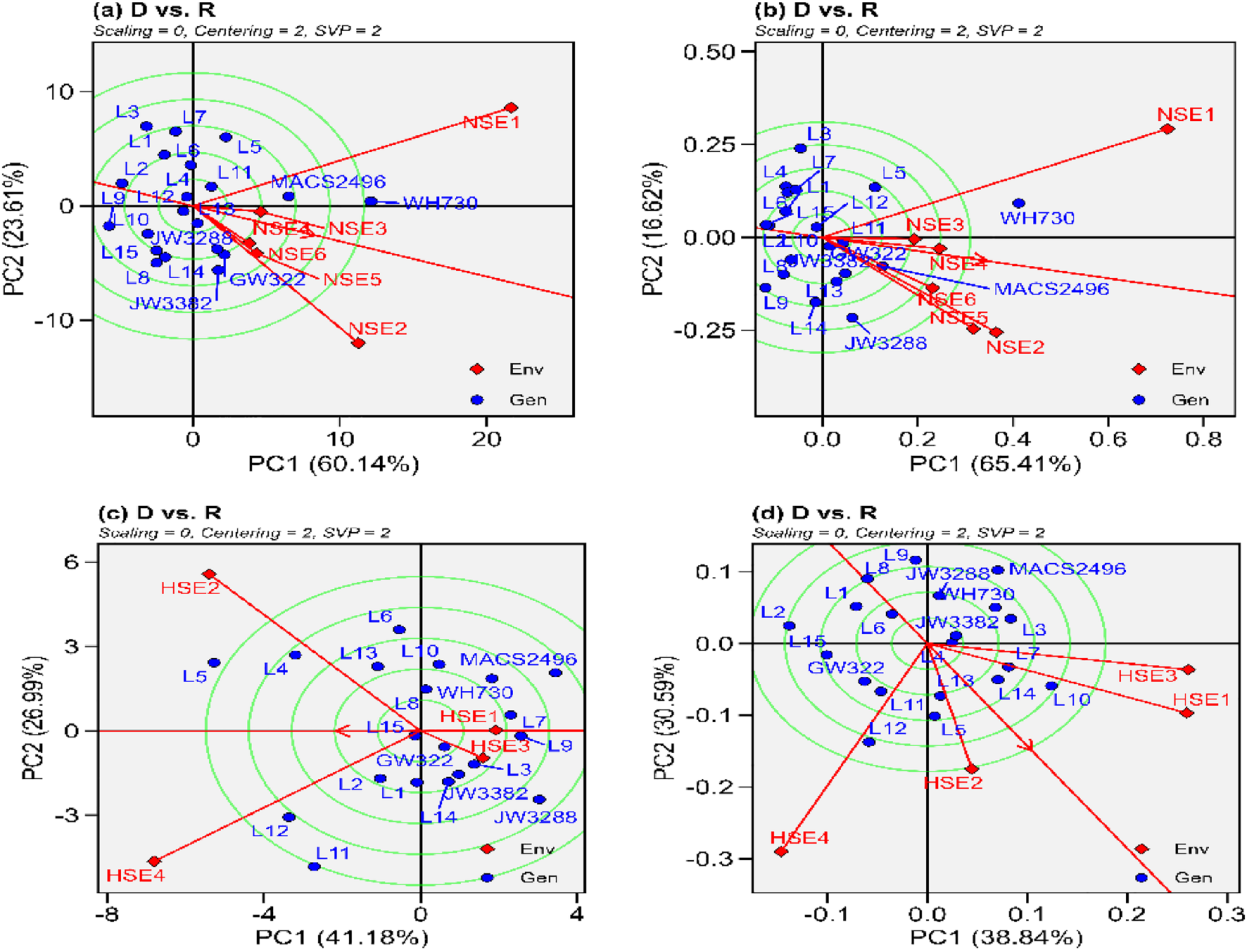
The “Discrimitiveness vs. Representativeness” model of biplot evaluate the wheat genotypes against the ideal genotypes, conferring genotype interaction and genotype x environment interactions. (**a**) grain yield under normal sown environment, (**b**) grain filling rate under normal sown environment, (**c**) grain yield under heat stress environment (**d**) grain filling rate under heat stress environment.

The GGE biplots for grain yield and grain filling rate of 20 selected heat tolerant wheat genotypes are shown in Figs. 3, 4, 5, and 6. As the x axis and y axis, the first and second principal component scores, respectively.

#### “Which-Won-Where” approaches

A "which-won-where" polygon view of the biplot showing which wheat genotype did best in which environment is shown in Fig. 3a to d and supplemental fig. s4. For grain yield and grain filling rate, respectively the biplots accounted variation approximately 83.75% and 82.03% (Fig. 3a and b) across normal sown environments, 68.17% and 69.43% (Fig. 3c and d) across heat stressed environments, 73.42% and 66.19% (Supplemental fig. s4) across all the environments from total variation related to genotype and GEI. The vertex wheat genotypes in each sector of the biplots represented the top-performing wheat genotypes in the environments that fell within that sector. Wheat genotypes that were positioned nearer the biplot's origin were more stable than vertex genotypes. The genotypes that formed the corners of the polygon for grain yield in the normal sown (L3, L5, WH730, GW322, L14, L9 and L2) (Fig. 3a) and in heat-stressed environments (L5, L6, MACS2496, JW3288, L11 and L12) (Fig. 3c) were utmost responsive genotypes to environments in their corresponding directions compared with the other genotypes. Similarly, for grain filling rate in normal sown environment (L3, WH730, JW3288, L9 and L15) (Fig. 3b) and in heat stressed environment (L2, L8, L9, MACS2496, L10 and L12) (Fig. 3d) genotypes were outmost responsive genotypes compared with other genotypes. The biplot was divided into several sectors by a line drawn perpendicular to the sides of the polygon and extending from the biplot's origin. For grain yield under normal sown environments, WH730 was the highest performing genotype at NSE1; JW3382 was the best genotype at NSE2, NSE3, NSE4, NSE5 and NSE6 (Fig. 3a), similarly for grain filling rate WH730 was the highest performing genotype at NSE1, NSE3 and NSE4; JW3288 was best at NSE2, NSE5 and NSE6 (Fig. 3b). Under heat stressed environments for grain yield, L5 at HSE2, L11 at HSE4, W3288 at HSE3 and MACS2496 at HSE1 were the winner genotypes (Fig. 3c) while for grain filling rate L10 was at HSE1, HSE2 and HSE3; L12 was at HSE4 identified as best performing genotypes, nevertheless L2, L8, L9 and MACS2496 did not win in any of the environments (Fig. 3d). Across all the environments for grain yield L5 was at HSE2; WH730 was at NSE1and NSE3; L2 was at HSE4; JW3382 was at NSE4, HSE3, HSE1, NSE6, NSE2 and NSE5 while for grain filling rate L3 was at HSE2; WH730 was at NSE5, NSE2, NSE6 and NSE3; L15 was at HSE4; L5 was at HSE3 and NSE1 identified as best performing genotypes (Supplemental fig. s4).

#### Mean vs. stability and ranking of genotypes

If single value portioning or SVP = 1, the biplot's origin is intersected by the average environment coordinate (AEC) line (single value portioning). The "Mean vs. stability" view often referred to as AEC and SVP, helps to simplify genotype assessment by focusing on mean performance and stability over different environmental conditions (Fig. 4). The biplot graph is made up of two straight lines: I the AEC ordinate (horizontal) and (ii) the AEC abscissa (vertical). The arrow on line one of Fig. 4 indicated in the direction of higher mean performance for the characteristic under this study. It can be seen that for grain yield and grain filling rate in normal sown environments WH730 (Fig. 4a and b) had the highest performing ability while L3 (Fig. 4a and b) had the lowest performing ability. Moreover, MACS2496 (Fig. 4a and b) had the highest performing stability, while L3 (Fig. 4a and b) had the lowest performing stability, and other cultivars had general stability in normal sown environments. In heat stressed environments for grain yield L5 (Fig. 4c) had the highest performing ability while MACS2496 (Fig. 4c) had the lowest performing ability. Whereas, for grain yield L2 (Fig. 4c) had the highest performing stability, while JW3288 (Fig. 4c) had the lowest performing stability in heat stress environments. Similarly, for grain filling rate L10 (Fig. 4d) had the highest performing ability while L2 (Fig. 4d) had the lowest performing ability in heat stress environments. Additionally, L4 (Fig. 4d) had the highest performing stability, while L2 (Fig. 4d) had the lowest performing stability. Across all the environments MACS2496 and L13 (Supplemental fig. s5) had the highest performing stability, while L3 and L9 (Supplemental fig. s5) had the lowest performing stability for grain yield and grain filling rate, respectively. The most stable genotypes for grain yield and grain filling rate were identified as JW3288 and L11 (Fig. 5a and b) in environments where seeds were sown timely, L15 and L13 (Fig. 5c and d) in heat-stressed environments, and L13 and L11 (Supplemental fig. s6) in both of these environmental conditions, respectively. These genotypes had above-average yields and were located on the AEC abscissa with zero (very low) projection onto the AEC ordinate. In contrast, L5 and L10 under heat stressed environment for grain yield and grain filling rate respectively (Fig. 5c and d), while for grain yield and grain filling rate WH730 under normal sown and across all the environmental conditions was identified as highest performing but less stable (Fig. 5a, b and supplemental fig. s6), as evident from greater projection onto the AEC ordinate.

#### Relationships, discrimination and representativeness of the test environments

Figure 6a to d and Supplemental fig. s7 present a vector view of the GGE biplot, elucidating the relationships between environmental interactions and the biplot origin through depicted vectors. All of the normal sown environments showed positive correlation (< 90°) for grain yield and grain filling rate (Fig. 6a and b). On the other hand, under heat stressed environments HSE1 and HSE3; HSE2 and HSE4 were positively correlated (Fig. 6c) for grain yield while for grain filling rate excluding HSE4 all the tested environments associated positively (Fig. 6d). Across all the environments for grain yield excluding HSE1 and HSE4 all the environments associated positively (Fig. S6) while for grain filling rate excluding HSE2 and HSE4 all the environments associated positively (Supplemental fig. s7). The "ideal environment" in Fig. 6 is represented by the circle at the centre of the concentric circle; it is a virtual environment with the longest vector (most discriminating) of all environments and is fully representative (i.e., it has no significant contribution to GEI and thus is positioned on the AEC abscissa). Similarly, for grain yield and grain filling rate, a cultivar closer to the centre has more stability, as shown in Fig. 6a to d, for grain yield L4 and L12, for grain filling rate L10 and L11 under normal sown environments while for grain yield L15 and GW322, for grain filling rate L4 and JW3382 in heat stressed environments had relatively high performing ability with good stability. Similarly, L12 for grain yield and grain filling rate has high performing ability and stability across all the environments (Supplement fig. s7).

## Discussion

In central India, a region heavily reliant on wheat for daily dietary needs, the identification of heat-tolerant, high-performing wheat varieties surpassing commercial checks is crucial for advancing nutritional security. Numerous studies have previously shown the potential for concurrent enhancement of wheat grain yield and grain filling rate under both heat stress and nonstress conditions^7,16,52^. The observed variations in grain yield and grain filling rate among the experimental genotypes, under both heat-stressed and non-stressed conditions, imply the possibility of identifying preferred genotypes that exhibit favourable traits across both environments. Prior investigations have documented significant variances among tested wheat genotypes concerning the examined traits in both heat-stressed and non-stress environments^40,53,54^. The discernible interactions observed between wheat genotype and environment, coupled with stability estimates derived from the univariate and multivariate stability analysis, imply the existence of a GEI interaction^55^. This suggests a dynamic alteration in the response patterns and ranking of wheat genotypes in response to diverse environmental conditions. Consistent findings were observed in prior research concerning wheat genotypes, encompassing both heat-stressed and non-stressed environments^40,56,57^. As indicated by the outcomes of preceding research, the environmental influences were notably significant across diverse environmental conditions^7,16,52,58^. As anticipated, environments subjected to heat stress demonstrated a diminished mean grain yield compared to non-stressed environments^2,4,41^.Previous reports in India have documented the grain yield superiority of newly developed recombinant lines over commercial checks^16,40,59^. This suggested that the newly created recombinant lines outperformed over the commercial checks under heat stress situations.

Elevated *bi* values observed in the majority of high-yielding and fast grain-filling wheat genotypes in this study suggest their heightened adaptability to high-yield environments^45^. As per Betrán et al.^60^, a positive correlation was observed between high mean performance and regression coefficient across diverse environmental conditions. According to univariate stability analysis, L12 and L11 exhibited the highest stability (low *S*^2^*d*_*i*_values) for grain yield and grain filling rate, respectively, under normal sown conditions. For both traits under heat stress, L3 and L6 displayed superior stability, while WH730 and L6 were identified as the most stable across all environments.

The predominance of genotype-environment interaction (GEI) over genotype suggests that the AMMI biplot is effective for visualizing genotype evaluation. Similar investigations were conducted in other crops to explore genotype-environment interaction (GEI) through the AMMI model^61–63^. Efficient selection of genotypes excelling in both stability and performance can be achieved through the Genomic Selection Index (GSI), calculated from AMMI stability values. Hence, JW3288, L8, and L13 emerged as superior genotypes for grain yield under normal sowing conditions, heat stress, and across all environments, respectively. This approach has been efficiently employed in other crops^64,65^. While AMMI can aid in selecting stable cultivars achieving both high and low yields, it often overlooks numerous high-yielding cultivars with poor stability^34,66^.

By segmenting the biplot, the GGE biplot provides a polygonal perspective, facilitating a clearer examination of 'which-won-where' patterns. "When diverse test environments cluster into distinct segments, it signifies the presence of diverse high-performing genotypes for those segments, indicating the existence of genotype-environment interaction (GEI)^66^. The polygonal view divided the biplot into two sectors: one represented by NSE1 and the other by all remaining environments, consistently observed across normal sown conditions for both grain yield and grain filling rate. In grain yield under heat stress, four sectors were identified across different environments, and for grain filling rate, two sectors represented by HSE3 and HSE4. Across all environments, both grain yield and grain filling rate exhibited two sectors represented by NSE1 and HSE. This data is essential for categorizing environments into distinct mega-environments and recommending specific wheat genotypes for each environment^21,67,68^. An optimal genotype, defined by Yan and Kang^69^ as one that excels in performance while maintaining stability. The most desired genotypes in this study were those that were closest to the ideal genotype in each environment. Across all of the test environments genotype L12 as the best genotype, telling this genotype's inherent capacity for superior performance and added adaptability. Yan^70,71^, declares that the association among the vectors of binary test environments is resolute via the cosine of the angle among them. Angles > 90° advised a negative association of genotype presentation among these environments, whereas lesser angles (< 90°) designated resemblance in genotype presentation among these environments. Right angles (90°) designated orthogonality and absence of association. It was obvious after the GGE biplot's vector interpretation that convinced environments had positive relationships while others showed negative relationships. While negative or low associations indicate considerable dissimilarity among environments resultant strongly influenced by Genotype-Environment Interaction (GEI) however, positive relationships suggest similarity in genotype performance across various environments^72,73^. When assessing crops, environments with long vectors and modest angles with the AEC abscissa are beneficial^66,74^.

## Conclusion

The present study identified the most promising stable heat tolerant wheat genotypes for the central India. Under various heat stress regimes L8, L15 and L1 were identified as most stable recombinant lines for grain yield while for grain filling rate L13, L5 and L4identified as most stable lines based on univariate stability analysis. Whereas, across heat stress and non-stressed environments GW322, L11, L12, and L13were identified as most stable lines for both the traits. When choosing outstanding wheat genotypes in terms of stability, the multivariate stability analysis performed better than the univariate stability model. The NSE3 and HSE2 identified as most stable environments. In heat stress regimes, L8 was identified as the most stable recombinant inbred line, determined through comprehensive analyses encompassing both univariate and multivariate stability assessments. The recombinant inbred line, L8, could be further tested and utilized in future breeding programs.

### Supplementary Information

Supplementary Information.

## Data Availability

The datasets of the current study can be requested from corresponding author with strong reason.

## References

[1] Pal, N., Saini, D.K. & Kumar, S. *Breaking yield ceiling in wheat: Progress and future prospects.* In Wheat (Intech.Open 2022).

[2] Gourdji SM, Sibley AM, Lobell DB (2013). Global crop exposure to critical high temperatures in the reproductive period: historical trends and future predictions. Environ. Res. Lett..

[3] Joshi AK, Mishra B, Chatrath R, Ortiz Ferrara G, Singh RP (2007). Wheat improvement in India: present status, emerging challenges and future prospects. Euphytica.

[4] Lobell DB, Sibley A, Ortiz-Monasterio JI (2012). Extreme heat effects on wheat senescence in India. Nat. Clim. Change.

[5] Pask A (2014). A wheat phenotyping network to incorporate physiological traits for climate change in South Asia. Field Crops Res..

[6] Sharma RC (2007). Wheat grain yield and stability assessed through regional trials in the Eastern Gangetic Plains of South Asia. Euphytica.

[7] Verma A, Chatrath R, Sharma I (2015). AMMI and GGE biplots for G×E analysis of wheat genotypes under rain fed conditions in central zone of India. J. Appl. Nat. Sci..

[8] Asseng S (2015). Rising temperatures reduce global wheat production. Nat. Clim. Chang..

[9] Lobell DB (2008). Prioritizing climate change adaptation needs for food security in 2030. Science.

[10] Mondal S (2013). Earliness in wheat: A key to adaptation under terminal and continual high temperature stress in South Asia. Field Crops Res..

[11] Rao BB, Chowdary PS, Sandeep VM, Pramod VP, Rao VUM (2014). Spatial analysis of the sensitivity of wheat yields to temperature in India. Agric. For. Meterol..

[12] Sonkar G (2019). Vulnerability of Indian wheat against rising temperature and aerosols. Environ. Pollut..

[13] Kumar SN (2014). Vulnerability of wheat production to climate change in India. Clim. Res..

[14] Hilmarsson HS, Rio S, Sanchez JIY (2021). Genotype by environment interaction analysis of agronomic spring barley traits in Iceland using AMMI, factorial regression model and linear mixed model. Agronomy.

[15] Khan MMH, Rafii MY, Ramlee SI, Jusoh M, Al-Mamun M (2021). AMMI and GGE biplot analysis for yield performance and stability assessment of selected Bambara groundnut (Vigna subterranea L Verdc) genotypes under the multi-environmental trials (METs). Sci. Rep..

[16] Singh C (2019). Genotype x environment interaction analysis of multi-environment wheat trials in India using AMMI and GGE biplot models. Crop Breed. Appl. Technol..

[17] Khazratkulova S (2015). Genotype environment interaction and stability of grain yield and selected quality traits in winter wheat in Central Asia. Turk. J. Agric. For..

[18] Tremmel-Bede K (2020). Stability analysis of wheat lines with increased level of arabinoxylan. PLoS ONE.

[19] Khazratkulova S (2015). Genotype × environment interaction and stability of grain yield and selected quality traits in winter wheat in Central Asia. Turk. J. Agric. For..

[20] Gauch HG (2013). A simple protocol for AMMI analysis of yield trials. Crop Sci..

[21] Gauch HG, Zobel RW (1997). Identifying mega-environments and targeting genotypes. Crop Sci..

[22] Modeling genotype-by-environment interaction and its genetic basis (2013). Malosetti, M., Ribaut, J.M. & VAN-Eeuwijk, F.A. The statistical analysis of multi-environment data. Front. Physiol..

[23] Myint KA (2019). Genetic diversity and selection criteria of MPOB Senegal oil palm (Elaeisguineensis Jacq.) germplasm by quantitative traits. Ind. Crops Prod..

[24] Gupta V (2023). AMMI and GGE biplot analysis of yield under terminal heat tolerance in wheat. Mol. Biol. Rep..

[25] Shahriari Z, Heidari B, Dadkhodaie A (2018). Dissection of genotype × environment interactions for mucilage and seed yield in Plantago species: Application of AMMI and GGE biplot analyses. PloS One.

[26] Elias AA, Robbins KR, Doerge RW, Tuinstra MR (2016). Half a century of studying genotype × environment interactions in plant breeding experiments. Crop Sci..

[27] Bocianowski J, Tratwal A, Nowosad K (2021). Genotype by environment interaction for main winter triticale varieties characteristics at two levels of technology using additive main effects and multiplicative interaction model. Euphytica.

[28] Bocianowski J, Warzecha T, Nowosad K, Bathelt R (2019). Genotype by environment interaction using AMMI model and estimation of additive and epistasis gene effects for 1000-kernel weight in spring barley (Hordeum vulgare L.). J. Appl. Genet..

[29] Yan W (2002). Singular-value partitioning in biplot analysis of multi environment trial data. Agron. J..

[30] Yan W, Kang MS, Ma B, Woods S, Cornelius PL (2007). GGE biplot vs AMMI analysis of genotype-by-environment data. Crop Sci..

[31] Sabaghnia N, Sabaghpour SH, Dehghani H (2008). The use of an AMMI model and its parameters to analyse yield stability in multi-environment trials. J. Agric. Sci..

[32] Sharifi P, Aminpanah H, Erfani R, Mohaddesi A, Abbasian A (2017). Evaluation of genotype × environment interaction in rice based on AMMI model in Iran. Rice Sci..

[33] Shrestha J, Subedi S, Acharya R, Sharma S, Subedi M (2021). Grain yield stability of maize (Zea mays L) hybrids using ammi model and GGE biplot analysis. SAARC J. Agric..

[34] Gauch HG, Piepho HP, Annicchiarico P (2008). Statistical analysis of yield trials by AMMI and GGE: Further considerations. Crop Sci..

[35] Bishnoi OP (2020). GGE biplot based stability analysis of durum wheat genotypes using statistical package GGE Biplot GUI. Int. J. Agric. Environ. Biotechnol..

[36] Mostafavi K, Imeni SH, Zare M (2011). Stability analysis of rice genotypes based GGE biplot Method in North of Iran. J. Appl. Sci. Res..

[37] Ruswandi D (2021). GGE biplot analysis for stability and adaptability of maize hybrids in western region of Indonesia. Int. J. Agron..

[38] Garg D, Sareen S, Dalal S, Tiwari R, Singh R (2012). Heat shock protein-based SNP marker for terminal heat stress in wheat ('triticumaestivum'L). Austral. J. Crop Sci..

[39] Kumar A, Kumar P, Singh G (2019). Assessment and role of genetic diversity of component traits for improving grain yield and heat tolerance in bread wheat (Triticum aestivum). Indian J. Agric. Sci..

[40] Sareen S, Tyagi BS, Sarial AK, Tiwari V, Sharma I (2014). Trait analysis, diversity, and genotype x environment interaction in some wheat landraces evaluated under drought and heat stress conditions. Chilean J. Agric. Res..

[41] Sharma D, Mamrutha HM, Gupta VK, Tiwari R, Singh R (2015). Association of SSCP variants of HSP genes with physiological and yield traits under heat stress in wheat. Res. Crops..

[42] Dias AS, Lidon FC (2009). Evaluation of grain filling rate and duration in bread and durum wheat, under heat stress after anthesis. J. Agronomy Crop Sci..

[43] Farshadfar E (2008). Incorporation of AMMI stability value and grain yield in a single non-parametric index (GSI) in bread wheat. Pak. J. Biol. Sci..

[44] Sandhu KS, Mihalyov PD, Lewien MJ, Pumphrey MO, Carter AH (2021). Combining genomic and phenomic information for predicting grain protein content and grain yield in spring wheat. Front. Plant Sci..

[45] Eberhart SA, Russell WA (1966). Stability parameters for comparing varieties 1. Crop Sci..

[46] Changizi M, Choukan R, Heravan EM, Bihamta MR, Darvish F (2014). Evaluation of genotype × environment interaction and stability of corn hybrids and relationship among univariate parametric methods. Can. J. Plant Sci..

[47] Fischer RA, Maurer R (1978). Drought resistance in spring wheat cultivars I. Grain yield responses. Austral. J. Agric. Res..

[48] Neisse AC, Kirch JL, Hongyu K (2018). AMMI and GGE biplot for genotype environment interaction: A medoid–based hierarchical cluster analysis approach for high–dimensional data. Biom. Lett..

[49] Rad MN (2013). Genotype environment interaction by AMMI and GGE biplot analysis in three consecutive generations of wheat (Triticum aestivum) under normal and drought stress conditions. Aust. J. Crop Sci..

[50] Yan W, Tinker NA (2006). Biplot analysis of multi-environment trial data: Principles and applications. Can. J. Plant Sci..

[51] Pour-Aboughadareh A, Khalili M, Poczai P, Olivoto T (2022). Stability indices to deciphering the genotype-by-environment interaction (GEI) effect: An applicable review for use in plant breeding programs. Plants.

[52] Amare K, Zeleke H, Bultosa G (2015). Variability for yield, yield related traits and association among traits of sorghum (Sorghum Bicolor (L) Moench) varieties in Wollo, Ethiopia. J. Plant Breed. Crop Sci..

[53] Qaseem MF, Qureshi R, Shaheen H (2019). Effects of pre-anthesis drought, heat and their combination on the growth, yield and physiology of diverse wheat (Triticum aestivum L.) genotypes varying in sensitivity to heat and drought stress. Sci. Rep..

[54] Rattey A, Shorter R, Chapman S, Dreccer F, Herwaarden AV (2009). Variation for and relationships among biomass and grain yield component traits conferring improved yield and grain weight in an elite wheat population grown in variable yield environments. Crop & Pasture Sci..

[55] Khan I (2023). Stability analysis of wheat through genotype by environment interaction in three regions of Khyber Pakhtunkhwa Pakistan. SABRAO J. Breed. Genet..

[56] Elbasyoni IS (2018). Performance and stability of commercial wheat cultivars under terminal heat stress. Agronomy.

[57] Kamara MM (2021). Combining ability and gene action controlling grain yield and its related traits in bread wheat under heat stress and normal conditions. Agronomy.

[58] Tembo B (2021). Genotype by environment interaction analysis of wheat (Triticum aestivum L.) grain yield under rainfed conditions in Zambia. SABRAO J. Breed Genet..

[59] Tanin MJ (2022). Ascertaining yield and grain protein content stability in wheat genotypes having the *Gpc-B1* gene using univariate, multivariate, and correlation analysis. Front Genet.

[60] Betrán FJ, Ribaut JM, Beck DL, Gonzalez-de LD (2003). Genetic analysis of inbred and hybrid grain yield under stress and non-stress environments. Crop Sci..

[61] Bashir EM, Ali AM, Ismail MI, Parzies HK, Haussmann BI (2014). Patterns of pearl millet genotype-by-environment interaction for yield performance and grain iron (Fe) and zinc (Zn) concentrations in Sudan. Field Crop Res..

[62] Nzuve F, Githiri S, Mukunya DM, Gethi J (2013). Analysis of genotype × environment interaction for grain yield in Maize hybrids. J. Agric. Sci..

[63] Vaezi B (2017). GGE biplot and AMMI analysis of barley yield performance in Iran. Cereal. Res. Commun..

[64] Donkor EF, Nyadanu D, Akromah R, Osei K (2020). Genotype-by environment interaction and stability of taro [Colocasia esculenta (l) Schott] genotypes for yield and yield components. Ecol Genet Genom..

[65] Nduwumuremyi A, Melis R, Shanahan P, Theodore A (2017). Interaction of genotype and environment effects on important traits of cassava (Manihot esculenta Crantz). Crop J..

[66] Yan W, Kang MS, Ma S, Woods B, Cornelius PL (2007). GGE biplot vs AMMI analysis of genotype-by-environment data. Crop Sci..

[67] Abakemal D, Shimelis H, John D (2016). Genotype-by environment interaction and yield stability of quality protein maize hybrids developed from tropical-highland an inbred line. Euphytica.

[68] Badu-Apraku B (2017). Yield gains in extra-early maize cultivars of three breeding eras under multiple environments. Agron. J..

[69] Yan W, Kang MS (2002). *GGE biplot analysis a graphical tool for breeders, geneticists, and agronomists* (CRC Press.

[70] Gasura E, Setimela P, Souta C (2015). Evaluation of the performance of sorghum genotypes using GGE biplot. Can. J. Plant Sci..

[71] Yan W (2002). Singular-value partitioning in biplot analysis of multi-environment trial data. Agron. J..

[72] Makumbi D, Diallo A, Kanampiu F, Mugo S, Karaya H (2015). Agronomic performance and genotype x environment interaction of herbicide-resistant maize varieties in eastern Africa. Crop Sci..

[73] Sserumaga JP (2016). Genotype by environment interactions and agronomic performance of doubled haploids testcross maize (Zea mays L) hybrids. Euphytica.

[74] Tukamuhabwa P, Assiimwe M, Nabasirye M, Kabayi P, Maphosa M (2012). Genotype by environment interaction of advanced generation soybean lines for grain yield in Uganda. Afr. Crop Sci. J..

