# Supplementary material for: Exploring the genotype-environment interaction of bread wheat in ambient and high-temperature planting conditions: a rigorous investigation
Source: Sci Rep. 2024 Jan 29;14:2402. doi: 10.1038/s41598-024-53052-w (PMC10825171; doi:10.1038/s41598-024-53052-w)
Supplement: Supplementary file 1 — Supplementary Information. [file 41598_2024_53052_MOESM1_ESM.pdf]

Exploring the genotype-environment interaction of bread wheat in ambient and high-temperature planting conditions: a rigorous investigation

Vikrant Khare [1,*], Rama Shankar Shukla [1], Suneeta Pandey [2], Sanjay Kumar Singh[1] and Charan Singh[3]

[1]Department of Plant Breeding and Genetics, Jawaharlal Nehru Krishi Vishwavidyalaya, Jabalpur, Madhya Pradesh, 482004, India, Email id: vikrantkhare@jnkvv.org*, *ORCID*: 0000000179704817*
[2]Department of Plant Breeding and Genetics, Agriculture University Jodhpur, Rajasthan, 342304, India
[3]Indian Institute of Wheat and Barley Research, 132001, Haryana, India

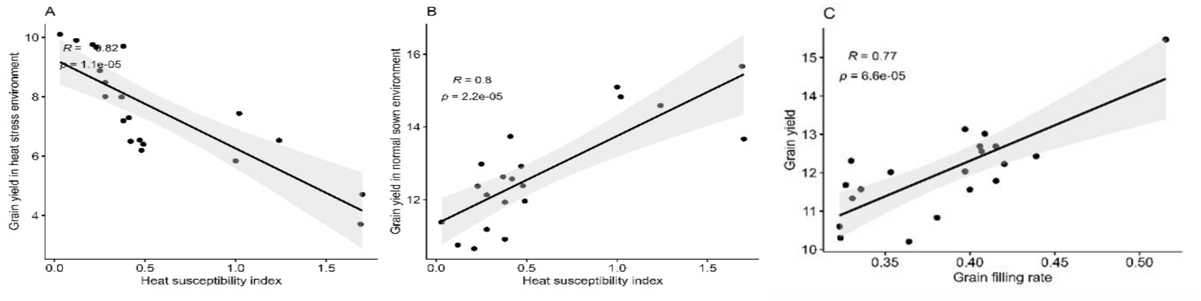

Supplemental Figure 1. Pattern of correlation coefficient A: correlation between heat susceptibility index and gain yield in heat stress environment, B: correlation between heat susceptibility index and gain yield in normal sown environment, C: correlation between grain filling rate and grain yield.

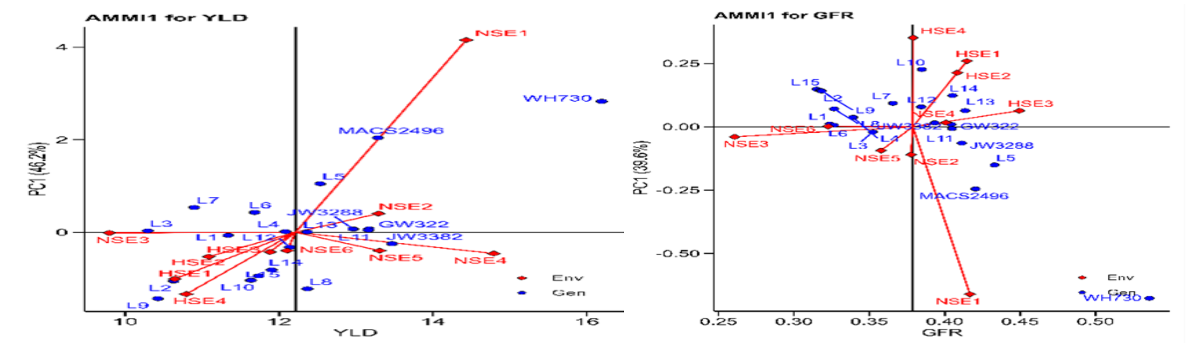

Supplemental Figure 2. Across all the environments “AMMI1” graphs displays the main effect and IPC1 effect values describing relationship among examined wheat genotype and environment.

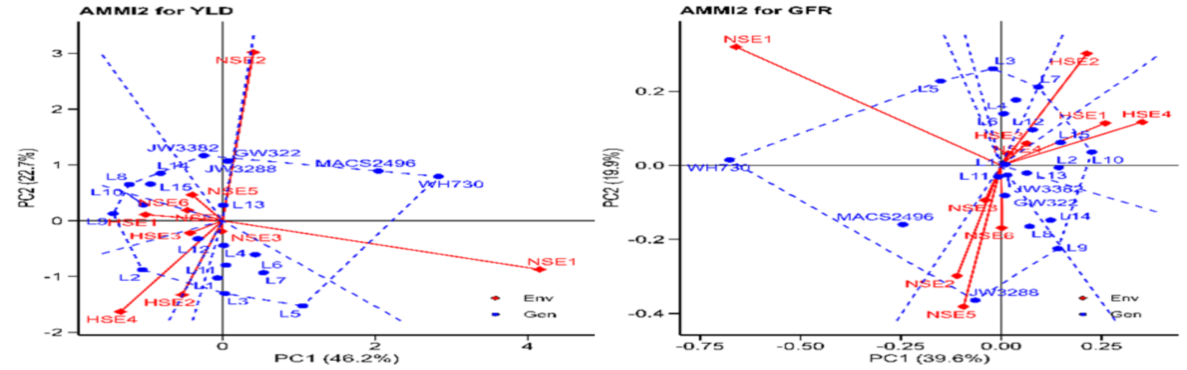

Supplemental Figure 3. Across all the environments “AMMI2” graphs displays both the axes of interaction (IPCA1 and IPCA2) values for genotype effect and genotype by environment interaction effect.

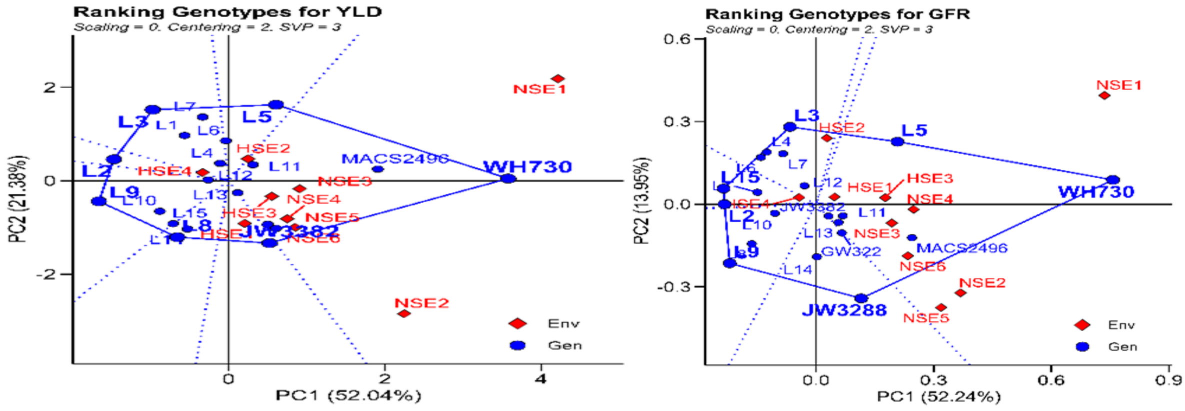

Supplemental Figure 4. Across all the environments polygon view of "Which-won-where" model of GGE biplot representing the performance of wheat genotypes and their interactions with environment.

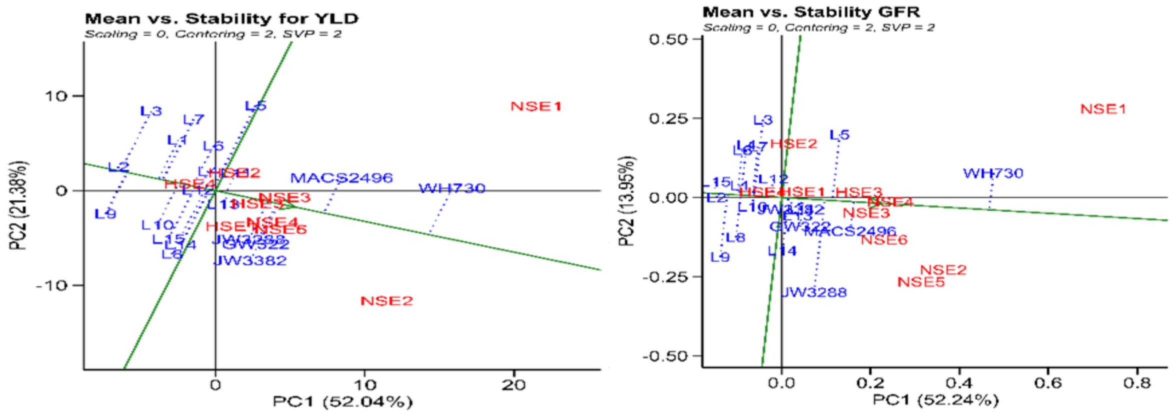

Supplemental Figure 5. Across all the environments "mean versus stability" model describing the interaction effect of wheat genotypes.

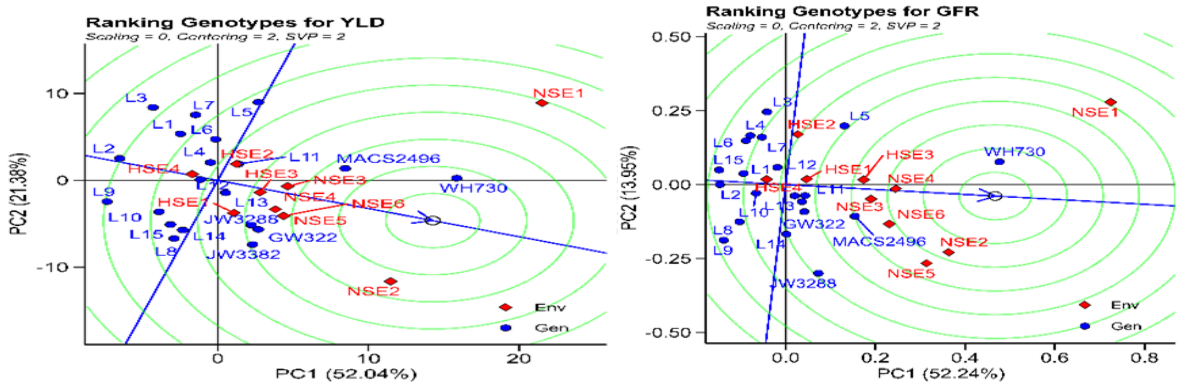

Supplemental Figure 6. Across all the environments "ranking genotypes" model of biplot assess other wheat genotypes against the ideal genotype conferring genotype interaction and genotype x environment interactions

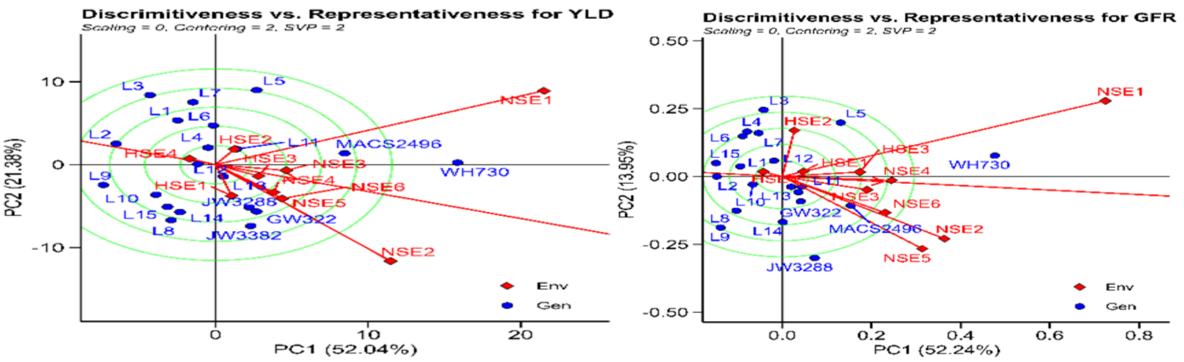

Supplemental Figure 7. Across all the environments "Discrimitiveness vs. Representativeness" model of biplot evaluate the wheat genotypes against the ideal genotypes, conferring genotype interaction and genotype x environment interactions.

Supplemental Table 1. Mean grain yield and grain filling rate for 20 genotypes evaluated in 6 normal sown environments.

ENV	GEN	YLD	GFR	ENV	GEN	YLD	GFR	ENV	GEN	YLD	GFR
NSE1	L1	14.20	0.36	NSE2	L1	8.80	0.30	NSE3	L1	9.40	0.30
NSE1	L2	9.10	0.26	NSE2	L2	8.05	0.23	NSE3	L2	10.50	0.28
NSE1	L3	13.50	0.48	NSE2	L3	6.55	0.15	NSE3	L3	12.00	0.27
NSE1	L4	14.20	0.41	NSE2	L4	13.65	0.27	NSE3	L4	11.00	0.21
NSE1	L5	20.50	0.65	NSE2	L5	10.55	0.36	NSE3	L5	7.90	0.25
NSE1	L6	15.90	0.39	NSE2	L6	11.85	0.34	NSE3	L6	10.80	0.29
NSE1	L7	16.40	0.40	NSE2	L7	8.55	0.28	NSE3	L7	7.60	0.21
NSE1	L8	8.80	0.26	NSE2	L8	14.80	0.37	NSE3	L8	10.10	0.24
NSE1	L9	6.50	0.19	NSE2	L9	9.10	0.29	NSE3	L9	7.50	0.21
NSE1	L10	9.40	0.29	NSE2	L10	12.90	0.37	NSE3	L10	9.10	0.22
NSE1	L11	16.90	0.47	NSE2	L11	11.30	0.37	NSE3	L11	8.90	0.28
NSE1	L12	13.70	0.43	NSE2	L12	13.10	0.41	NSE3	L12	7.30	0.22
NSE1	L13	14.40	0.40	NSE2	L13	15.30	0.48	NSE3	L13	7.60	0.18
NSE1	L14	10.10	0.31	NSE2	L14	15.80	0.49	NSE3	L14	8.00	0.23
NSE1	L15	9.50	0.29	NSE2	L15	15.60	0.32	NSE3	L15	9.10	0.17
Parents											
NSE1	WH730	29.40	1.03	NSE2	WH730	20.10	0.61	NSE3	WH730	15.00	0.42
NSE1	MACS2496	23.20	0.55	NSE2	MACS2496	17.30	0.55	NSE3	MACS2496	10.00	0.33
Commercial checks											
NSE1	GW322	15.00	0.43	NSE2	GW322	18.20	0.47	NSE3	GW322	10.40	0.25
NSE1	JW3382	13.50	0.40	NSE2	JW3382	18.40	0.46	NSE3	JW3382	12.40	0.31
NSE1	JW3288	14.50	0.38	NSE2	JW3288	16.00	0.45	NSE3	JW3288	11.30	0.40

Supplemental Table 1. Continued …

ENV	GEN	YLD	GFR	ENV	GEN	YLD	GFR	ENV	GEN	YLD	GFR
NSE4	L1	11.75	0.26	NSE5	L1	12.65	0.29	NSE6	L1	11.45	0.28
NSE4	L2	13.95	0.45	NSE5	L2	12.05	0.29	NSE6	L2	10.85	0.28
NSE4	L3	13.25	0.38	NSE5	L3	9.90	0.27	NSE6	L3	8.70	0.29
NSE4	L4	13.25	0.28	NSE5	L4	11.65	0.26	NSE6	L4	10.45	0.28
NSE4	L5	13.90	0.45	NSE5	L5	13.10	0.39	NSE6	L5	11.90	0.37
NSE4	L6	14.65	0.31	NSE5	L6	10.40	0.24	NSE6	L6	9.20	0.18
NSE4	L7	14.65	0.47	NSE5	L7	10.55	0.27	NSE6	L7	9.35	0.22
NSE4	L8	13.65	0.31	NSE5	L8	14.80	0.35	NSE6	L8	13.60	0.36
NSE4	L9	13.55	0.33	NSE5	L9	15.00	0.40	NSE6	L9	13.80	0.38
NSE4	L10	16.20	0.44	NSE5	L10	12.60	0.31	NSE6	L10	11.40	0.34
NSE4	L11	15.95	0.45	NSE5	L11	15.30	0.43	NSE6	L11	14.10	0.35
NSE4	L12	15.20	0.37	NSE5	L12	13.65	0.35	NSE6	L12	12.45	0.23
NSE4	L13	15.00	0.48	NSE5	L13	13.20	0.38	NSE6	L13	12.00	0.41
NSE4	L14	14.05	0.38	NSE5	L14	13.75	0.39	NSE6	L14	12.55	0.40
NSE4	L15	14.55	0.32	NSE5	L15	12.10	0.24	NSE6	L15	10.90	0.29
Parents											
NSE4	WH730	18.00	0.56	NSE5	WH730	16.65	0.54	NSE6	WH730	15.45	0.55
NSE4	MACS2496	13.80	0.46	NSE5	MACS2496	15.45	0.50	NSE6	MACS2496	14.25	0.27
Commercial checks											
NSE4	GW322	17.50	0.49	NSE5	GW322	13.45	0.43	NSE6	GW322	12.25	0.34
NSE4	JW3382	19.65	0.49	NSE5	JW3382	13.10	0.28	NSE6	JW3382	11.90	0.34
NSE4	JW3288	13.35	0.39	NSE5	JW3288	16.80	0.60	NSE6	JW3288	15.60	0.37

ENV = Environment, *GEN* = Genotypes, *YLD* = Grain yield, *GFR* = Grain filling rate, *NSE1* = Jabalpur-Dece-2019-20, *NSE2* = Sagar-Dece-2019-20, *NSE3* = Narmadapuram-Dece-2019-20, *NSE4* = Jabalpur-Dece-2020-21, *NSE5* = Sagar-Dece-2020-21, *NSE6* = Narmadapuram-Dece-2020-21.

Supplemental Table 2. Mean grain yield and grain filling rate for 20 genotypes evaluated across 4 heat stress environments.

ENV	GEN	YLD	GFR	ENV	GEN	YLD	GFR
HSE1	L1	9.80	0.31	HSE2	L1	10.30	0.36
HSE1	L2	9.75	0.31	HSE2	L2	10.15	0.35
HSE1	L3	9.35	0.49	HSE2	L3	9.10	0.41
HSE1	L4	8.70	0.39	HSE2	L4	14.35	0.46
HSE1	L5	8.35	0.39	HSE2	L5	15.15	0.54
HSE1	L6	10.50	0.36	HSE2	L6	13.25	0.48
HSE1	L7	10.75	0.51	HSE2	L7	10.10	0.43
HSE1	L8	12.65	0.36	HSE2	L8	12.30	0.38
HSE1	L9	9.55	0.39	HSE2	L9	9.15	0.32
HSE1	L10	13.35	0.65	HSE2	L10	12.05	0.44
HSE1	L11	11.45	0.42	HSE2	L11	10.15	0.36
HSE1	L12	9.50	0.40	HSE2	L12	11.00	0.45
HSE1	L13	10.35	0.43	HSE2	L13	13.10	0.53
HSE1	L14	10.40	0.42	HSE2	L14	9.90	0.41
HSE1	L15	11.75	0.35	HSE2	L15	11.15	0.42
Parents							
HSE1	WH730	12.40	0.42	HSE2	WH730	11.80	0.41
HSE1	MACS2496	9.40	0.41	HSE2	MACS2496	10.10	0.37
Commercial checks							
HSE1	GW322	12.65	0.43	HSE2	GW322	10.45	0.42
HSE1	JW3382	11.70	0.46	HSE2	JW3382	10.00	0.35
HSE1	JW3288	10.55	0.41	HSE2	JW3288	8.20	0.30

Supplemental Table 2. Continued …

ENV	GEN	YLD	GFR	ENV	GEN	YLD	GFR
HSE3	L1	13.40	0.42	HSE4	L1	11.65	0.38
HSE3	L2	9.85	0.32	HSE4	L2	12.05	0.43
HSE3	L3	10.55	0.49	HSE4	L3	10.05	0.30
HSE3	L4	12.05	0.50	HSE4	L4	11.55	0.35
HSE3	L5	10.95	0.50	HSE4	L5	13.05	0.44
HSE3	L6	10.85	0.38	HSE4	L6	9.45	0.31
HSE3	L7	12.15	0.51	HSE4	L7	8.90	0.37
HSE3	L8	12.60	0.35	HSE4	L8	10.40	0.30
HSE3	L9	11.30	0.41	HSE4	L9	8.80	0.28
HSE3	L10	9.65	0.45	HSE4	L10	9.70	0.37
HSE3	L11	12.40	0.44	HSE4	L11	15.15	0.51
HSE3	L12	11.10	0.44	HSE4	L12	14.40	0.56
HSE3	L13	12.20	0.45	HSE4	L13	10.50	0.41
HSE3	L14	13.35	0.61	HSE4	L14	11.15	0.43
HSE3	L15	11.55	0.34	HSE4	L15	11.15	0.43
Parents							
HSE3	WH730	14.10	0.53	HSE4	WH730	9.05	0.30
HSE3	MACS2496	12.20	0.53	HSE4	MACS2496	7.15	0.25
Commercial checks							
HSE3	GW322	10.90	0.35	HSE4	GW322	10.90	0.45
HSE3	JW3382	13.00	0.47	HSE4	JW3382	11.05	0.38
HSE3	JW3288	13.55	0.48	HSE4	JW3288	9.80	0.35

ENV = Environment, *GEN* = Genotypes, *YLD* = Grain yield, *GFR* = Grain filling rate, *HSE1* = Jabalpur-Jan-2019-20, *HSE2* = Narmadapuram-Jan-2019-20, *HSE3* = Jabalpur-Jan-2020-21, *HSE4* = Narmadapuram-Jan-2020-21.

Supplemental Table 3. Across all the environment stability parameters for grain yield and grain filling rate

Genotypes	YLD							GFR						
	G_m	R_{gm}	b_i	S^2d_i	ASV	R_{ASV}	GSI	G_m	R_{gm}	b_i	S^2d_i	ASV	R_{ASV}	GSI
L1	11.80	9	0.98	2.38	1.30	9	18	0.32	17	0.53	0.00	0.02	1	18
L2	10.84	16	0.44	2.31	0.87	5	21	0.32	19	0.35	0.00	0.29	12	31
L3	10.56	18	0.42	4.83	1.75	15	33	0.35	13	1.41	0.01	0.26	10	23
L4	12.29	8	0.71	1.04	0.46	2	10	0.34	14	1.45	0.00	0.19	8	22
L5	13.15	2	2.00	7.16	3.21	20	22	0.43	2	1.58	0.01	0.38	16	18
L6	12.58	5	1.19	4.49	1.97	16	21	0.33	16	0.97	0.00	0.14	6	22
L7	11.62	10	1.83	4.98	2.64	19	29	0.37	12	1.87	0.00	0.28	11	23
L8	12.97	3	0.58	9.70	2.09	18	21	0.33	15	0.35	0.00	0.22	9	24
L9	11.39	11	1.07	7.42	2.02	17	28	0.32	18	0.49	0.01	0.36	15	33
L10	11.36	12	1.22	2.51	1.48	12	24	0.39	10	1.41	0.01	0.45	18	28
L11	12.51	7	1.67	5.21	1.36	10	17	0.41	7	0.83	0.00	0.03	2	9
L12	12.54	6	1.85	0.90	0.76	4	10	0.38	11	1.41	0.00	0.18	7	18
L13	13.55	1	1.61	3.47	1.27	8	9	0.41	4	1.35	0.00	0.13	5	9
L14	12.78	4	1.38	4.47	1.71	14	18	0.41	6	1.23	0.01	0.29	13	19
L15	11.02	15	1.16	4.38	1.65	13	28	0.32	20	0.99	0.00	0.30	14	34
Parents														
WH730	11.10	14	0.78	0.05	0.20	1	15	0.54	1	0.9	0.04	1.34	20	21
MACS2496	08.92	20	0.40	2.17	1.22	7	27	0.42	3	1.11	0.01	0.51	19	22
Commercial checks														
GW322	11.14	13	0.58	3.46	1.44	11	24	0.40	8	0.84	0.00	0.08	4	12
JW3382	10.57	17	0.44	0.52	0.60	3	20	0.39	9	0.93	0.00	0.04	3	12
JW3288	10.22	19	0.20	0.89	1.21	6	25	0.41	5	0	0.01	0.39	17	22

YLD = Grain yield, *GFR* = Grain filling rate, $G_{m=}$ grand mean, R_{gm} = Rank by grand mean, b_i = Regression coefficient of Eberhart and Russell, S^2d_i= Deviation form regression of Eberhart and Russel model, *ASV* = AMMI stability value, R_{ASV} = Rank by AMMI stability value, *GSI* = Genomic selection index.

Supplemental Table 4. Across all the environments AMMI-based ANOVA for grain yield and grain filling rate

Source	YLD				GFR		
	DF	MSS	Percent Explained	*p*-value	MSS	Percent Explained	*p*-value
Environment	9	54.20	12.3	<0.001	0.118	14.5	<0.001
Block	10	0.50	0.001	0.65	0.001	0.002	0.143
Genotype	19	26.60	12.8	<0.001	0.059	15.4	<0.001
G x E	171	8.29	35.8	<0.001	0.014	33.7	<0.001
PC1	27	18.30	46.2	<0.001	0.036	39.6	<0.001
PC2	25	12.40	22.7	<0.001	0.019	19.9	<0.001
PC3	23	8.68	14.1	<0.001	0.012	11.4	<0.001
PC4	21	7.70	11.4	<0.001	0.010	8.1	<0.001
PC5	19	6.14	8.20	<0.001	0.009	6.9	<0.001
PC6	17	4.33	5.20	<0.001	0.008	5.5	<0.001
PC7	15	2.78	2.90	<0.001	0.006	3.9	<0.001
PC8	13	1.52	1.40	<0.001	0.006	2.9	<0.001
PC9	11	0.14	0.10	<0.001	0.004	1.9	<0.001
Residuals	190	0.65	2.10	-	0.0009	0.02	-
Total	570	6.94	-	-	7.32	-	-

YLD = Grain yield, *GFR*= Grain filling rate, *DF*= degree of freedom, *MSS*= mean sum of square, *G x E* = Genotype by environment interactions, *PC* = Principal component, $*p < 0.05$, $**p < 0.01$ and $***p < 0.001$.

Supplemental Table 5. Meteorological data for Jabalpur district in Madhya Pradesh, India, was collected on a weekly basis during the crop growth period.

Period	Temperature (^{0}C)				Relative Humidity				Rainfall		Rainy days		Sun Shine hours	
	Maximum		Minimum		Morning	Morning	Evening	Evening	(milli meters)	(milli meters)	(in days)	(in days)	(hrs day^{-1})	(hrs day^{-1})
	2019-20	2020-21	2019-20	2020-21	2019-20	2020-21	2019-20	2020-21	2019-20	2020-21	2019-20	2020-21	2019-20	2020-21
3Dec.-9Dec.	25.8	29.3	8.4	9.1	90	81	46	29	0	0	0	0	6.8	9.4
10-16Dec.	24.8	26.4	12.1	14.7	95	88	70	57	12.4	2.3	2	0	3.6	3.2
17-23Dec.	21	23.2	6.3	5.5	89	74	55	31	0	0	0	0	5.3	7.4
24-31Dec.	22.1	23.8	6.7	7.2	87	83	50	43	0	0	0	0	5.9	6.2
1-7Jan.	20.3	26.5	10.3	12.4	91	87	62	50	17.9	0.5	2	0	3.5	4.5
8-14Jan.	22	25	8	11.8	89	86	48	49	15.8	0.4	1	0	7.7	5.7
15-21Jan.	23.8	25.4	11.2	8	89	75	51	31	0.4	0	0	0	5.9	8.2
22-28Jan.	24.1	24.6	9.1	8.6	84	86	44	49	0	0	0	0	7.8	7.4
29Jan.-4Feb.	24.2	21.4	8.3	4.8	88	73	41	31	0	0	0	0	7.3	6
5-11Fab.	22.2	26.4	7.9	8.9	85	72	40	34	8.7	0	1	0	6.6	9.1
12-18Fab.	26.8	27.7	7.5	11.8	90	83	32	42	0	12.6	0	1	9.8	7.7
19-25Fab.	28.8	28.6	14.1	10.6	86	79	49	28	0	0	0	0	6.4	9.3
26Fab. -4Mar.	29.2	32.7	12.7	12.4	87	74	34	25	1.8	0	0	0	8.3	9.8
5-11Mar.	28.5	34.8	13.1	13	84	74	45	20	0.8	0	0	0	7.4	8.9
12-18Mar.	29.9	32.3	14.7	15.3	81	78	35	29	0.5	6.2	0	1	7.6	7
19-25Mar.	31.6	33.9	16.9	16.7	69	67	40	27	36.4	0	1	0	7.2	5
26Mar.-1Apr.	34.5	36.8	20.4	16.7	61	57	33	13	0.9	0	0	0	7.5	8.5
2-8Apr.	36.8	38.3	20.3	14.8	45	64	22	11	0	0	0	0	7.1	8.6
9-15Apr.	38.9	37.7	21.6	18.6	37	61	21	22	0	0.6	0	0	9.4	8
16-22Apr.	38.7	37	24.5	18	45	53	23	19	0	0	0	0	7.7	7.2
23-29Apr.	37.9	38.2	23	17.5	51	52	28	13	0	0	0	0	7.8	9.9
30Apr.-6May	39.3	38.8	24	23.2	50	47	26	24	0	2.6	0	1	10	7.8

Dec. = December, *Jan.* = January, *Feb.* = February, *Mar.* = March, *Apr.* = April, *May* = May, *hrs day^{-1}* = Hours per day.

Supplemental Table 6. Meteorological data for Narmadapuram district in Madhya Pradesh, India, was collected on a weekly basis during the crop growth period.

Period	Temperature (° C)				Relative humidity (%)				Rainfall		Rainy days		Sun Shine	
	Maximum		Minimum		Morning		Evening		(milli meters)	(milli meters)	(in days)	(in days)	(hrs day^{-1})	(hrs day^{-1})
	2019-20	2020-21	2019-20	2020-21	2019-20	2020-21	2019-20	2020-21	2019-20	2020-21	2019-20	2020-21	2019-20	2020-21
3 Dec.-9 Dec.	30	30.5	10	11	80	76	33	45	0	0	0	0	4.4	8.5
10 Dec.-16 Dec.	29.5	31.5	11.5	10.5	94	71	59	40	8	0	1	0	7.2	6.7
17 Dec.-23 Dec.	27	28.5	10	16.5	93	96	42	53	0	7.2	0	4	7.1	6.6
24 Dec.-31 Dec.	26.5	25.5	5.5	8.5	82	80	42	39	1.8	0	1	0	8.2	4.3
1 Jan.-7 Jan.	25	26	7	9	77	65	52	40	2	0	1	0	6.9	7.3
8 Jan.-14 Jan.	27	26	4	13.5	94	90	47	57	0	1.5	0	1	4.8	7.7
15 Jan.-21 Jan.	27	27	5	7	93	92	59	41	0	0	0	0	7.8	8.9
22 Jan.-28 Jan.	27	28.5	7.5	6	96	80	48	39	0	0	0	0	3.3	9.6
29 Jan.-4 Feb.	24.5	27.5	6.5	7	82	93	47	45	0	0	0	0	8	9.3
5 Feb.- 11 Feb.	24.5	29	6.5	5.5	96	74	38	31	0	0	0	0	6.4	8.1
12 Feb.-18 Feb.	30	28	7	5.5	87	84	35	42	0	0	0	0	7.2	9
19 Feb.-25 Feb.	31	28	8.5	6	80	88	30	40	0	1	0	1	7.5	9.1
26 Feb.-4 Mar.	30	32	9	11	73	74	34	28	1.2	0	1	0	7.3	9.2
4 Mar.- 11 Mar.	30	35	10	11	80	66	27	14	0	0	0	0	7.9	7.7
12 Mar.-18 Mar.	33	35.5	9	11.5	68	75	23	14	0	0	0	0	6	6.7
19 Mar.-25 Mar.	39.2	37	12	14	74	78	30	33	3.1	1.8	1	1	8.1	7.7
26 Mar.-1 Apr.	39.5	36	17	14	93	66	20	25	8.5	0	2	0	8	8.4
2 Apr.-8 Apr.	41.2	40.2	15	14.5	54	62	15	9	0	0	0	0	7.5	8.7
9 Apr.-15 Apr.	44.3	40.5	17.5	15	46	51	18	9	0	0	0	0	8.1	7.3
16 Apr.-22 Apr.	42.8	41	18	22	57	50	16	12	2.8	0	1	0	9.2	8
23 Apr.-29 Apr.	40.3	40.5	20.5	21	52	49	16	14	0	0	0	0	9.4	9.5
30 Apr.-6 May	43	40.5	22	22.5	48	54	17	11	0	0	0	0	9.2	9.1

Dec. = December, *Jan.* = January, *Feb.* = February, *Mar.* = March, *Apr.* = April, *May* = May, *hrs day*$^{-1}$ = Hours per day.

Supplemental Table 7. Meteorological data for Sagar district in Madhya Pradesh, India, was collected on a weekly basis during the crop growth period.

Period	Temperature (° C)				Relative humidity (%)				Rainfall		Rainy days		Sun Shine	
	Maximum		Minimum		Morning		Evening		(milli meters)	(milli meters)	(in days)	(in days)	(hrs day^{-1})	(hrs day^{-1})
	2019-20	2020-21	2019-20	2020-21	2019-20	2020-21	2019-20	2020-21	2019-20	2020-21	2019-20	2020-21	2019-20	2020-21
3 Dec.-9 Dec.	25.1	26.9	12.4	11.2	68.1	82	54.1	68	0	0	0	0	7.8	8.1
10 Dec.-16 Dec.	23.9	25.7	13.2	15.7	85.6	87	68	66	0.7	0.3	1	1	4.6	4.2
17 Dec.-23 Dec.	22	22.6	9.2	8.1	83.6	85	61	70	0	0	0	0	5.3	7.7
24 Dec.-31 Dec.	20.1	22.8	6.9	6.9	78.4	81	60.1	67	0	0	0	0	5.7	6.5
1 Jan.-7 Jan.	21.5	27.2	9.4	10.8	95.6	87	76.9	79	0	0	0	0	3.7	4.5
8 Jan.-14 Jan.	22.4	24.4	9.3	9.1	73.6	80	55.1	77	0.6	0.8	1	1	4.7	4.3
15 Jan.-21 Jan.	22.6	26.3	12.1	6.7	87.9	82	69.7	70	0.2	0	1	0	6.2	7.2
22 Jan.-28 Jan.	23.3	26.8	9.4	8.8	75	83	53.7	75	1	0	1	0	4.8	7.4
29 Jan.-4 Feb.	23.5	28.4	11.5	7.4	64.6	79	44.3	65	0	0	0	0	7.1	6
5 Feb.- 11 Feb.	23.1	27.0	10.4	8.9	63.6	77	46.7	69	0.6	0	1	0	4.6	8.8
12 Feb.-18 Feb.	28.1	27.9	11.6	12.1	53.6	82	35.9	73	0	0	0	0	8.8	7.7
19 Feb.-25 Feb.	30	30.4	15.9	13.4	54.4	74	33.3	64	0	0	0	0	6.4	9.5
26 Feb.-4 Mar.	29.2	31.4	15.3	14.1	68.7	64	39.1	53	0	0	0	0	8.1	9.8
4 Mar.- 11 Mar.	29.6	33.1	15.9	16.1	61.3	55	33.6	45	0.6	0	1	0	7.4	8.9
12 Mar.-18 Mar.	29.3	34.2	15.6	17.1	62	70	36	56	3.2	0	2	0	6.6	7
19 Mar.-25 Mar.	33	32.9	18.7	18.8	53.7	61	32.4	47	0	0	0	0	6.9	5
26 Mar.-1 Apr.	32.5	37.3	19.7	16.9	56.1	43	42.3	32	3.8	0	2	0	7.5	8.5
2 Apr.-8 Apr.	36.8	37.9	20.7	17.2	39.1	42	26.4	31	0	0	0	0	7.1	8.6
9 Apr.-15 Apr.	38.2	38.9	23.6	22.2	33.4	45	20	36	0	0	0	0	9.6	8
16 Apr.-22 Apr.	39.6	40.1	25.4	21.1	34.9	46	25.9	35	0	0	0	0	7.7	7.2
23 Apr.-29 Apr.	42.3	42.2	24.3	23.3	35.3	35	26.8	31	0	0	0	0	7.8	9.3
30 Apr.-6 May	42.1	40.3	25.6	24.1	33.1	42	24.3	35	0	0	0	0	9.4	7.8

Dec. = December, *Jan.* = January, *Feb.* = February, *Mar.* = March, *Apr.* = April, *May* = May, *hrs* day^{-1} = Hours per day.
